# Active template rotaxane synthesis through the Ni-catalyzed cross-coupling of alkylzinc reagents with redox-active esters†
†Electronic supplementary information (ESI) available. See DOI: 10.1039/c9sc02457c


**DOI:** 10.1039/c9sc02457c

**Published:** 2019-06-19

**Authors:** Javier Echavarren, Malcolm A. Y. Gall, Adrian Haertsch, David A. Leigh, Vanesa Marcos, Daniel J. Tetlow

**Affiliations:** a School of Chemistry , University of Manchester , Oxford Road , Manchester , M13 9PL , UK . Email: david.leigh@manchester.ac.uk

## Abstract

The synthesis of unsymmetrical axle [2]rotaxanes through a recently developed Ni-catalyzed C(sp^3^)–C(sp^3^) cross-coupling of redox-active esters (formed directly from carboxylic acids) and organozinc reagents (derived from alkyl bromides) is reported. The method also furnishes, as a minor product, the symmetrical axle [2]rotaxanes resulting from the homo-coupling of the organozinc half-thread. The rotaxanes are formed in up to 56% yield with the ratio of unsymmetrical rotaxane increasing with the cavity size of the macrocycle. In the absence of the redox-active ester neither rotaxane is formed, even though the homo-coupling rotaxane product does not incorporate the redox-active ester building block. A Ni(iii) intermediate is consistent with these observations, providing support for the previously postulated mechanism of the Ni-catalyzed cross-coupling reaction.

## Abstract 2

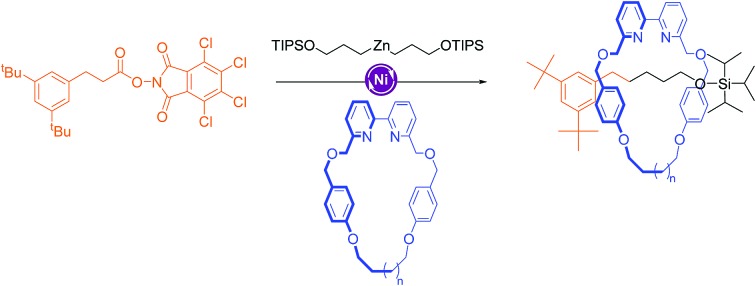
The Ni-catalyzed C(sp^3^)–C(sp^3^) cross-coupling of redox-active esters and organozinc reagents is used for the active template synthesis of ‘impossible’ rotaxanes.

## Introduction

Active template synthesis^1^ exploits coupling reactions (typically metal-promoted but recently expanded to include metal-free systems^2^) that are accelerated through a macrocycle or loop to afford a mechanically interlocked product (rotaxane,^3^–^5^ catenane^6^ or knot^7^). The strategy has several key differences to traditional ‘passive template’ interlocked molecule synthesis,^8^ including avoiding the need for intercomponent recognition motifs that persist in the threaded product.^9^ Most active template reactions still result in new functional groups in the rotaxane axle (*e.g.* the CuAAC cycloaddition, the most widely employed active template reaction,^3^ which produces a triazole ring), but the Ni-catalyzed C(sp^3^)–C(sp^3^) homo-coupling of alkyl bromides,^5^ where the connection between the thread building blocks results in a C–C bond within an alkyl chain,^4^ does not. This type of rotaxane is sometimes referred to as an ‘impossible’^10^ (or ‘improbable’^11^) rotaxane, as the axle in the final product does not contain an obvious template nor a retrosynthetic disconnection that seemed feasible prior to the development of traceless rotaxane synthetic strategies.^5^,^10^

Aside from the conceptual interest in constructing structural- and functional-group-minimalist interlocked systems, rotaxane assembly through simple C–C bond-forming reactions allows for molecular designs free of groups that are superfluous to the function of the final structure.^5^,^10^ However, the homo-coupling of the original active template Ni-catalyzed reaction restricts its use to the synthesis of rotaxanes with symmetric axles.^5^ In recent years homogeneous nickel catalysis has experienced considerable progress,^12^ particularly with respect to alkyl–alkyl cross-coupling methods.^13^ In 2016, Baran and co-workers reported a Ni-catalyzed C(sp^2^)–C(sp^3^) decarboxylative cross-coupling between activated carboxylic acids, in the form of redox-active esters, and arylzinc reagents derived from aryl bromides.14*a* The versatility of this strategy rapidly led to the development of a range of different coupling transformations,^14^ including a C(sp^3^)–C(sp^3^) version using redox-active esters and alkylzinc species.14*b* Given its general applicability and usefulness in molecular construction, we explored the reaction's utility for active template rotaxane synthesis.

## Results and discussion

Developing active template synthesis from a transition metal catalyzed reaction is far from straightforward:^1^ first, the metal has to stay coordinated to the macrocycle at—and between—crucial stages of the mechanism (during which the oxidation state of the ion may change several times) in order for axle formation to be directed through the cavity. Second, the macrocycle design must ensure that the metal coordinates *endo*-, not *exo*-, to the cavity and that the axle building blocks bind through opposite faces of the ring in order that their coupling leads to rotaxane formation. Finally, the conformation of the macrocycle needs to be such that the coordinated coupling reaction is favoured through the cavity rather than the generally less sterically demanding route to the side.

**Scheme 1:**
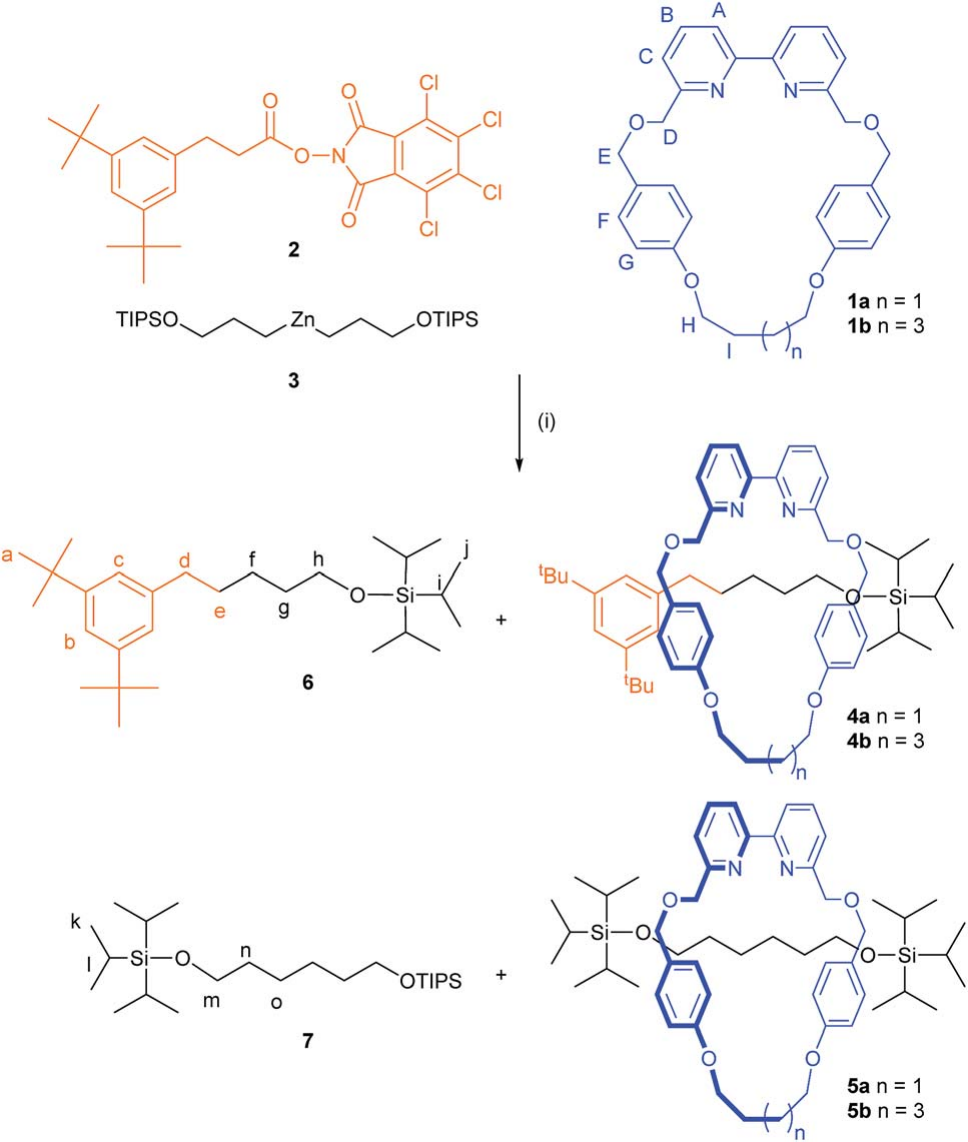
Synthesis of [2]rotaxanes by active template Ni-promoted coupling of a redox-active ester (**2**) and organozinc reagent (**3**). Reagents and conditions: (i) **2** (1.0 or 5.0 equiv.), **3** (2.0 or 10.0 equiv.; obtained from the corresponding alkyl bromide), **1a** or **1b** (1.0 equiv.), NiCl_2_·glyme (50 mol%), THF/DMF, r.t., 18 h. TIPS = triisopropylsilyl.

The Ni-catalyzed C(sp^3^)–C(sp^3^) decarboxylative cross-coupling is reported to work well with either 2,2′-bipyiridine or 4,4′-di-*tert*-butyl-2,2′-bipyridine ligands,14*b* but it was unclear whether a 6,6′-substitution pattern, which could direct a coordinated Ni-center towards the cavity of macrocycle **1a**, would be tolerated by the reaction. We were pleased to find that cross-coupling of redox-active ester **2** and organozinc compound **3** (obtained from the corresponding alkyl bromide *via* the Grignard reagent, see ESI†) using macrocycle **1a** as a ligand afforded unsymmetrical axle rotaxane **4a** in an initial 16% yield (Scheme 1 and Table 1, entry 1). In addition to **4a**, the symmetric [2]rotaxane, **5a**, which must arise through homo-coupling of the alkylzinc reagent **3**, was formed in 9% yield alongside non-interlocked threads **6** and **7**. When the reaction was carried out using a macrocycle with a slightly larger cavity, **1b**, the same level of conversion to rotaxane species was obtained but with an increase in the amount of cross-coupling rotaxane **4b** (20%) relative to the homo-coupling product **5b** (5%, Table 1, entry 2). Upon increasing the ratio of the axle building blocks to macrocycle, the amount of interlocked products increased (39%, Table 1, entry 3). While the small cavity macrocycle **1a** gave the rotaxanes in a close-to-1 : 1 ratio (**4a** 19% and **5a** 20%, Table 1, entry 3), the use of the larger macrocycle (**1b**) resulted in a 56% conversion with roughly 2 : 1 ratio in favour of the unsymmetrical rotaxane (**4b** 38% and **5b** 18%, Table 1, entry 4). The reaction could be scaled up five-fold without affecting the level of conversion (0.25 mmol scale, **4b** 33% and **5b** 13%, Table 1, entry 5). Changing the amount of catalyst correlated linearly with the formation of the free axle, **6**. However there was no significant impact on rotaxane formation; doubling the loading of NiCl_2_·glyme to 100 mol% resulted in 46% conversion (**4b** 35% and **5b** 11%, Table 1, entry 6), while reducing it to 25 mol% led to 39% conversion (**4b** 21% and **5b** 18%, Table 1, entry 7).

**Table 1:** Active template rotaxane formation under different reaction conditions^*a*^

Entry	Macrocycle	Equiv. **2**	Equiv. **3**	Rotaxane yield [%] (**4a** : **5a**)^*b*^	Rotaxane yield [%] (**4b** : **5b**)^*b*^
1	**1a**	1.0	2.0	25 (16 : 9)	—
2	**1b**	1.0	2.0	—	25 (4 : 1)
3	**1a**	5.0	10.0	39 (19 : 20)	—
4	**1b**	5.0	10.0	—	56 (19 : 9)
5^*c*^	**1b**	5.0	10.0	—	46 (33 : 13)^*d*^
6^*e*^	**1b**	5.0	10.0	—	46 (35 : 11)
7^*f*^	**1b**	5.0	10.0	—	39 (7 : 6)
8	**1a**	0.0	10.0	0	—
9	**1b**	0.0	10.0	—	0

^*a*^Unless otherwise stated, 0.05 mmol (1.0 equiv.) of **1a** or **1b** and 0.025 mmol NiCl_2_·glyme (50 mol%) were used.

^*b*^Ratios based on the integration of rotaxane and macrocycle ^1^H NMR signals.

^*c*^Reaction performed at a 0.25 mmol scale with respect to **1b** (1.0 equiv.).

^*d*^Isolated yield and ratios.

^*e*^100 mol% of NiCl_2_·glyme.

^*f*^25 mol% of NiCl_2_·glyme.

We repeated similar reactions in the absence of redox-active ester **2** with the intention of obtaining exclusively the symmetric rotaxanes **5a**/**b**, however, only the homo-coupling free thread **7** was formed under such conditions, with no interlocked species observed (Table 1, entries 8 and 9). To our initial surprise, the presence of the redox-active ester **2** appears to be essential not only for the formation of the cross-coupling rotaxanes **4a**/**b**, but also for the homo-coupling of half-thread **3** to take place through the cavity of the macrocycle.

**Fig. 1:**
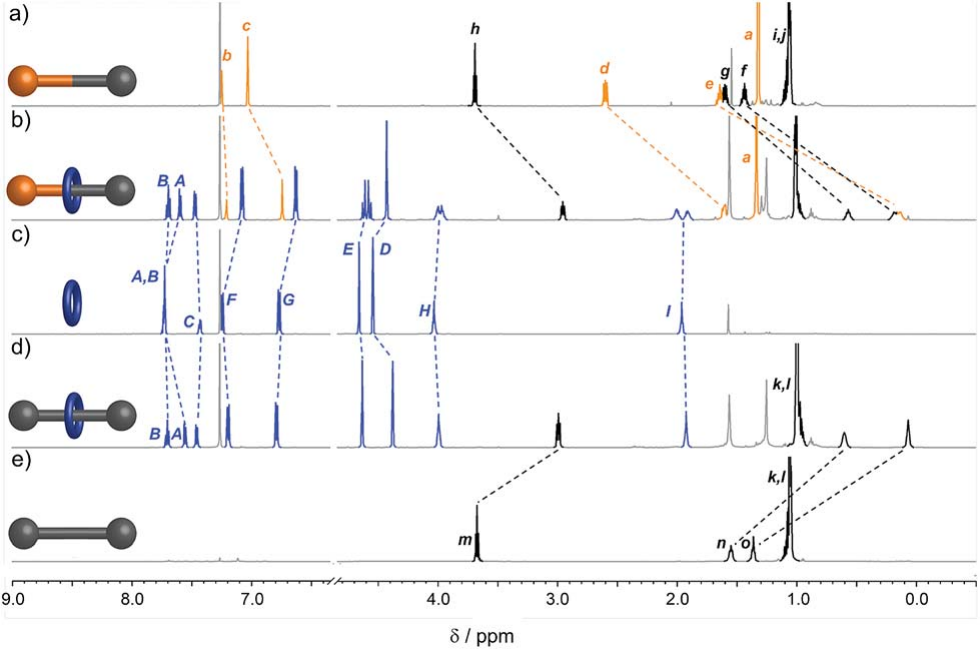
^1^H NMR spectra (600 MHz, CDCl_3_, 298 K) of (a) unsymmetrical thread **6**, (b) rotaxane **4a**, (c) macrocycle **1a**, (d) rotaxane **5a**, and (e) symmetrical thread **7**. Assignments correspond to the labelling shown in Scheme 1.

The interlocked structure of rotaxanes **4a** and **5a** was confirmed by mass spectrometry and comparison of the ^1^H NMR spectra with those of the non-interlocked macrocycle **1a** and threads **6** and **7** (Fig. 1). In the rotaxanes the signals from the aliphatic chain in the axle are shifted upfield due to the shielding effect of the aromatic rings of the macrocycle (Fig. 1b and d). The effect is less pronounced for resonances closer to the bulky stopper groups as the macrocycle's access to this region is sterically restricted. The two faces of the macrocycle experience different chemical environments in rotaxane **4a** because of the unsymmetrical axle, resulting in *H*_E_, *H*_H_ and *H*_I_ appearing as diastereotopic signals (Fig. 1b). In contrast, the faces of the macrocycle in symmetrical rotaxane **5a** are equivalent (Fig. 1d).

**Scheme 2:**
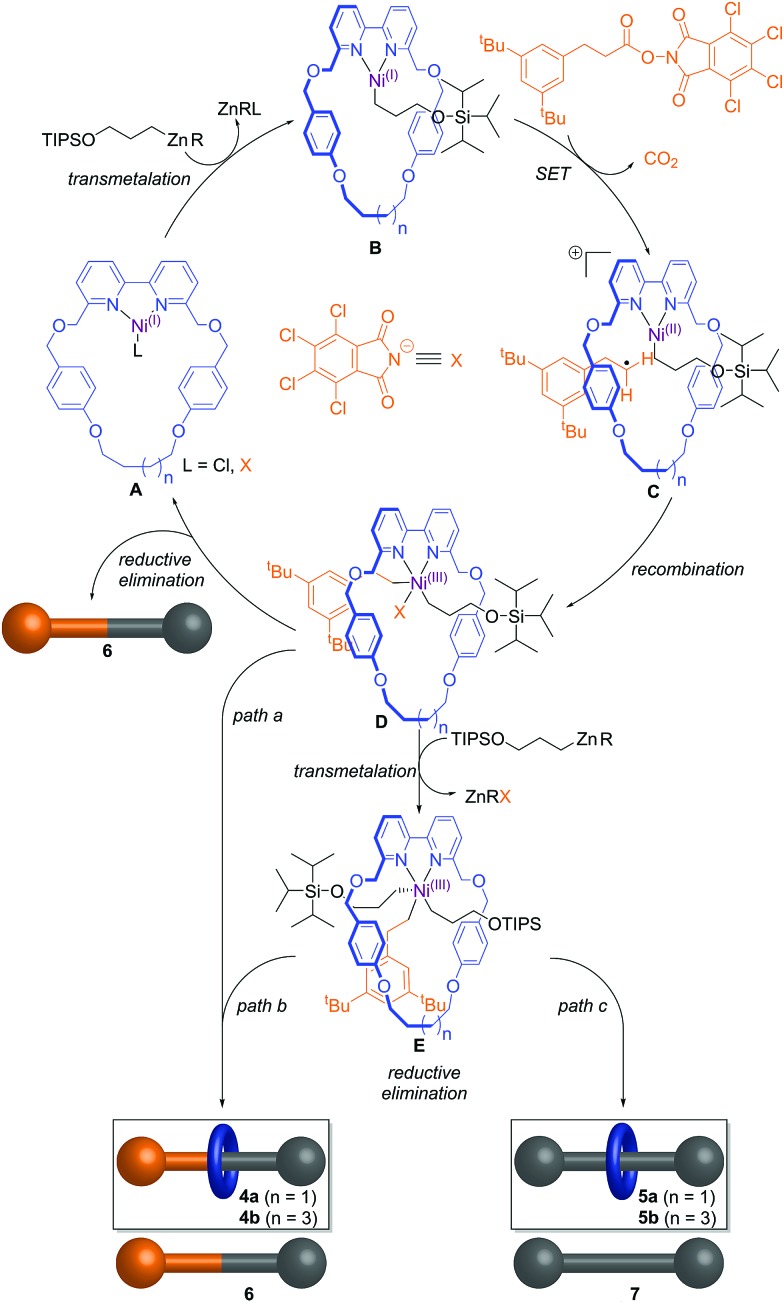
Proposed mechanistic cycle for the active template synthesis of rotaxanes through the Ni-promoted coupling of redox-active ester (**2**) and organozinc reagent (**3**). The key intermediate Ni(iii) **D** leads to non-interlocked thread **6** if the reductive elimination occurs outside the macrocycle cavity or to rotaxane **4a**/**b** if it takes place through the cavity. Alternatively, intermediate **D** could undergo another transmetalation event to form complex **E** from which either rotaxane **4a**/**b** or **5a**/**b** and non-interlocked thread **6** or **7** is obtained.

The catalytic cycle likely commences14*b* by transmetallation of the alkylzinc unit **3** with a slowly formed Ni(i) complex **A** to form intermediate **B** (Scheme 2). Single electron transfer (SET) from the Ni centre to the phthalimide group of the redox-active ester followed by decarboxylation forms a primary alkyl radical and cationic intermediate **C** that after radical recombination generates Ni(iii) intermediate **D**. This species can undergo reductive elimination^15^ to afford the non-interlocked thread **6**, in turn regenerating the Ni(i) species **A**. If the reductive elimination happens through the cavity of the macrocycle, the intermediate **D** instead leads to the formation of the cross-coupling rotaxane **4a**/**b** (path a).

From entries 8 and 9 in Table 1, the redox-active ester is required for the homo-coupling event to take place through the cavity of the macrocycle. This can be rationalized by a second transmetalation event of Ni(iii) intermediate **D** with organozinc unit **3**,^16^ which would lead to intermediate **E**. The arrangement of the ligands around the Ni centre in **E** will determine the outcome of the product after the reductive elimination, forming either the cross-coupling rotaxane **4a**/**b** and free thread **6** (path b) or the homo-coupling rotaxane **5a**/**b** and the corresponding non-interlocked thread **7** (path c). Thus the experimental observations are consistent with, and supportive of, the original Ni(iii)-intermediate mechanism postulated14*b* by Baran and co-workers.

## Conclusions

6,6′-Substituted bipyridine macrocycles enable the active template synthesis of unsymmetrical alkyl chain axle rotaxanes through the Ni-catalyzed decarboxylative C(sp^3^)–C(sp^3^) cross-coupling of redox-active ester and organozinc building blocks derived from carboxylic acids and alkyl bromides. Rotaxane formation, including that of a minor homo-coupling product, is consistent with—and supportive of—the Ni(iii) intermediate mechanism originally suggested for the coupling reaction. The combination of coordination assembly and catalysis inherent to active metal template synthesis is not only a useful tool for making functional-group-minimalist molecular structures, but can also provide experimental evidence3b,^5^ with regards to reaction mechanism.

## Conflicts of interest

There are no conflicts to declare.

## Supplementary Material

Supplementary information

## References

[cit1] Crowley J. D., Goldup S. M., Lee A.-L., Leigh D. A., McBurney R. T. (2009). Chem. Soc. Rev..

[cit2] De Bo G., Dolphijn G., McTernan C. T., Leigh D. A. (2017). J. Am. Chem. Soc..

[cit3] Aucagne V., Hänni K. D., Leigh D. A., Lusby P. J., Walker D. B. (2006). J. Am. Chem. Soc..

[cit4] Berna J., Crowley J. D., Goldup S. M., Hänni K. D., Lee A.-L., Leigh D. A. (2007). Angew. Chem., Int. Ed..

[cit5] Goldup S. M., Leigh D. A., McBurney R. T., McGonigal P. R., Plant A. (2010). Chem. Sci..

[cit6] Goldup S. M., Leigh D. A., Long T., McGonigal P. R., Symes M. D., Wu J. (2009). J. Am. Chem. Soc..

[cit7] Barran P. E., Cole H. L., Goldup S. M., Leigh D. A., McGonigal P. R., Symes M. D., Wu J., Zengerle M. (2011). Angew. Chem., Int. Ed..

[cit8] (a) Molecular Catenanes, Rotaxanes and Knots, ed. J.-P. Sauvage and C. O. Dietrich-Buchecker, Wiley-VCH, Weinheim, 1999.

[cit9] Bruns C. J. and Stoddart J. F., The Nature of the Mechanical Bond, John Wiley & Sons, Inc., Hoboken, NJ, USA, 2016.

[cit10] Hannam J. S., Lacy S. M., Leigh D. A., Saiz C. G., Slawin A. M. Z., Stitchell S. G. (2004). Angew. Chem., Int. Ed..

[cit11] Riss-Yaw B., Clavel C., Laurent P., Waels P., Coutrot F. (2018). Chem.–Eur. J..

[cit12] Tasker S. Z., Standley E. A., Jamison T. F. (2014). Nature.

[cit13] Choi J., Fu G. C. (2017). Science.

[cit14] Cornella J., Edwards J. T., Qin T., Kawamura S., Wang J., Pan C.-M. J., Gianatassio R., Schmidt M., Eastgate M. D., Baran P. S. (2016). J. Am. Chem. Soc..

[cit15] Bour J. R., Camasso N. M., Meucci E. A., Kampf J. W., Canty A. J., Sandford M. S. (2016). J. Am. Chem. Soc..

[cit16] Zheng B., Tang F., Luo J., Schultz J. W., Rath N. P., Mirica L. M. (2014). J. Am. Chem. Soc..

