# Supplementary material for: Active template rotaxane synthesis through the Ni-catalyzed cross-coupling of alkylzinc reagents with redox-active esters
Source: Chem Sci. 2019 Jun 19;10(30):7269–73. doi: 10.1039/c9sc02457c (PMC6686731; doi:10.1039/c9sc02457c)
Supplement: Supplementary file 1 [file SC-010-C9SC02457C-s001.pdf]

Electronic Supplementary Material (ESI) for Chemical Science.
This journal is © The Royal Society of Chemistry 2019

-Supporting Information-

Active Template Rotaxane Synthesis Through the Ni-Catalyzed Cross-Coupling of Alkylzinc Reagents with Redox-Active Esters

Javier Echavarren, Malcolm A. Y. Gall, Adrian Haertsch, David A. Leigh*, Vanesa Marcos, Daniel J. Tetlow

School of Chemistry, University of Manchester

Oxford Road, Manchester, M13 9PL, UK

E-mail: david.leigh@manchester.ac.uk

Table of Contents

S1. General Methods and Abbreviations 3
S1.1 General methods 3
S1.2 Abbreviations 3
S2. General Procedures and Synthesis 4
S2.1 General procedure for synthesis of organozinc **3** 4
S2.2 General procedure for rotaxane formation 5
S2.3 Procedure for preparative scale synthesis of rotaxanes 6
S2.4 Synthesis of organozinc precursor **S1** 7
S2.5 Synthesis of macrocycle **1b** 8
S2.6 Synthesis of redox-active ester **2** 11
S2.7 Synthesis of cross-coupling free thread **6** 12
S2.8 Synthesis of homo-coupling free thread **7** 14
S2.9 Synthesis of rotaxanes **4a**, **4b**, **5a**, and **5b** 15
S3. ^{1}H NMR Stackplots of Rotaxanes 18
S4. ^{1}H and ^{13}C NMR Spectra 19
S5. References 29

S1. General Methods and Abbreviations

S1.1 General methods

Unless stated otherwise, all reagents and solvents were purchased from commercial sources and used without further purification. Anhydrous DMF, CH_2Cl_2 and THF were obtained by passing the solvent (HPLC grade) through a Phoenix SDS System made by JC Meyers Solvent Systems. Flash column chromatography was carried out using Aldrich Si 60 Å (particle size 40–63 µm, Sigma Aldrich, UK) as the stationary phase. Analytical TLC was performed on pre-coated Merck silica gel plates (0.25 mm thickness, 60 F254) and observed under a 254 nm UV light or stained with a potassium permanganate solution. Preparative TLC separations were carried out using precoated Merck silica gel plates (1000 µm to 250 µm thickness, 60 F254). NMR spectra were recorded on a Bruker Avance III equipped with a cryoprobe (5 mm CPDCH 13C–1H/D) with an Oxford AS 600 magnet. ^{1}H NMR spectra were recorded at 600 MHz and ^{13}C spectra at 151 MHz. Chemical shifts are reported from high to low frequency in parts per million (ppm) relative to residual $CHCl_3$ (δH = 7.26, δC = 77.0) as the internal standard. All ^{1}H NMR resonances are reported to the nearest 0.01 ppm, and all ^{13}C resonances are reported to the nearest 0.01 ppm. Coupling constants are reported in hertz (Hz) and are reported to the nearest 0.1 Hz. Standard abbreviations indicating multiplicity were used as follows: s = singlet, d = doublet, t = triplet, q = quartet, m = multiplet and br = broad. ^{1}H NMR assignments were made using 2D NMR methods (COSY, HSQC and HMBC). Low resolution ESI mass spectrometry was performed with a Thermo Scientific LCQ Fleet Ion Trap Mass Spectrometer or an Advion Expression LCMS single quadrupole MS detector. High-resolution mass spectrometry was carried out by the EPSRC National Mass Spectrometry Service Centre (Swansea, UK) or by staff at the Mass Spectrometry Service, School of Chemistry, University of Manchester. Melting points were determined using a Büchi M–565 apparatus and are corrected. Macrocycle **1a** was prepared according to a literature procedure.[1]

S1.2 Abbreviations

DMAP, 4-dimethylaminopyridine. DMF, dimethyl formamide. EDCI, 1-Ethyl-3-(3-dimethylaminopropyl)carbodiimide. EDTA, ethylenediaminetetra-acetic acid. Na_4EDTA, ethylenediaminetetraacetic acid tetrasodium salt. $NiCl_2$·glyme, Nickel(II) chloride ethylene glycol dimethyl ether complex. PE, petroleum ether. Tf_2O, trifluoromethanesulfonic anhydride. THF, tetrahydrofuran. TIPS, triisopropylsilyl. TIPSCl, triisopropylsilyl chloride.

S2. General Procedures and Synthesis

S2.1 General procedure for synthesis of organozinc **3**

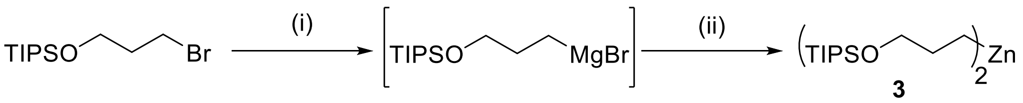

Scheme S1. Synthesis of organozinc reagent **3**. Reagents and conditions: (i) Mg, I_2, r.t., 90 min. (ii) $ZnCl_2$ (0.5 M in THF), 0 °C to r.t.

An oven-dried microwave vial equipped with a stirrer bar was charged with Mg turnings (0.54 g, 22.5 mmol, 1.5 equiv.) and I_2 (38 mg, 0.15 mmol, 0.01 equiv.). The vial was purged with N_2 before a solution of alkyl bromide **S1** (4.43 g, 15.0 mmol, 1.0 equiv.) in THF (23 mL, 0.65 M) was added. After 5 min the solution turned colourless and was left stirring at room temperature for 90 min. An aliquot was titrated over a solution of I_2 in THF to give a yield of around 60% (corresponding to a Grignard concentration of 0.39 M). In a separate vial equipped with a stirrer bar and flushed with N_2, a 0.5 M solution of $ZnCl_2$ in THF (2.5 mmol, 5.0 mL) was added, and the solution was cooled down to 0 °C, after which the 0.39 M solution Grignard reagent was added (12.9 mL, 5.0 mmol). The reaction was stirred and allowed to reach room temperature after which the organozinc solution **3** was ready to be used.

S2.2 General procedure for rotaxane formation

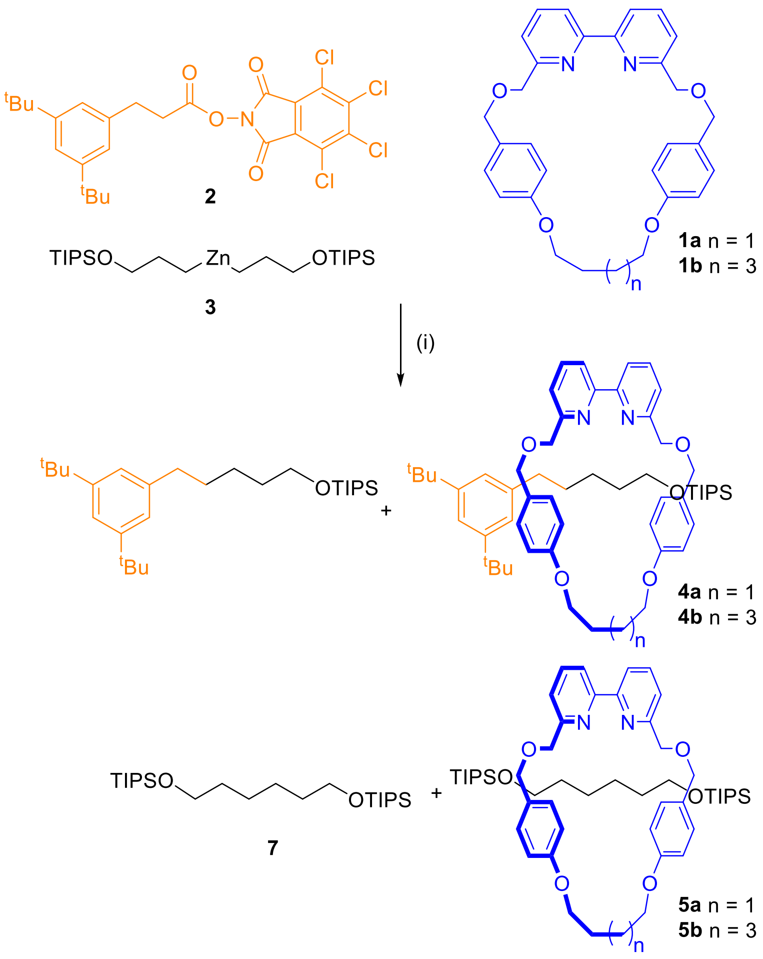

Scheme S2. Reagents and conditions: (i) $NiCl_2$·glyme, DMF, r.t., 5 min, then THF, r.t., 18 h.

An oven-dried microwave vial equipped with a stir bar was charged with redox-active ester **2** (1.0 equiv. or 5.0 equiv.) and the vial was purged with N_2. A solution of macrocycle **1a** or **1b** (0.05 mmol, 1.0 equiv.) and $NiCl_2$·glyme (5.4 mg, 0.025 mmol, 0.5 equiv.) stirred under N_2 in DMF (1.0 mL) for 5 min was added to the vial containing the redox-active ester **2**. The mixture was stirred for 1 min before the corresponding amount of organozinc solution **3** in THF (twice the equivalents of redox-active ester **2**) was added. The solution was left stirring at room temperature for 18 h. The reaction was quenched with a saturated aqueous solution of NH_4Cl and diluted with CH_2Cl_2 and the solution was washed with a saturated aqueous solution of Na_4EDTA. The aqueous phase was extracted with CH_2Cl_2 twice and the combined organic phases were further washed with water and then with brine. The organic phase was dried over Na_2SO_4, filtered and concentrated under reduced pressure and the crude was placed under high vacuum for 30 min before analysis by ^1H NMR. The conversion was calculated by comparing the ratio of free macrocycle **1a** or **1b** to rotaxane by ^1H NMR in $CDCl_3$. The crude mixture was purified by flash chromatography (SiO_2, *n*-hexane/EtOAc 6:1) to collect both rotaxanes (homodimer and heterodimer) as a mixture. Further separation by preparative thin-layer chromatography (1000 μm, 2 elutions, CH_2Cl_2) afforded the heterodimer as the upper band and the homodimer as the lower band.

S2.3 Procedure for preparative scale synthesis of rotaxanes

An oven-dried round bottom flask equipped with a stir bar was charged with redox-active ester **2** (1.25 mmol, 681 mg, 5.0 equiv.) and the flask was purged with N_2. A solution of macrocycle **1a** or **1b** (0.25 mmol, 128 mg, 1.0 equiv.) and $NiCl_2$·glyme (0.125 mmol, 27.5 mg, 0.5 equiv.) stirred under N_2 in DMF (5 mL) for 5 min was added to the flask containing the redox-active ester **2**. The mixture was stirred for 1 min before the freshly prepared organozinc solution **3** in THF (2.5 mmol, 18 mL, 10.0 equiv.) was added. The solution was left stirring at room temperature for 18 h. The reaction was quenched with a saturated aqueous solution of NH_4Cl (10 mL), diluted with CH_2Cl_2 and the solution was washed with a saturated aqueous solution of Na_4EDTA. The aqueous phase was extracted with CH_2Cl_2 twice and the combined organic phases were further washed with water and brine. The organic phase was dried over Na_2SO_4, filtered and concentrated under reduced pressure and the crude was placed under high vacuum for 30 min before analysis by ^1H NMR. The yield was calculated by comparing the ratio of free macrocycle **1a** or **1b** to rotaxane by ^1H NMR in $CDCl_3$. The determined NMR yields were 30 % for the heterodimer and 13 % for the homodimer. The crude mixture was purified by flash chromatography (SiO_2, n-hexane/EtOAc 6:1) to collect both rotaxanes (homodimer and heterodimer) as a mixture with further impurities. Purification by GPC in $CHCl_3$/0.1% Et_3N and preparative thin-layer chromatography (1000 µm, 1 elution, CH_2Cl_2) afforded the heterodimer (77 mg, 33 %) as the upper band (R_f = 0.5) and the homodimer (30 mg, 13 %) as the lower band (R_f = 0.3).

S2.4 Synthesis of organozinc precursor **S1**

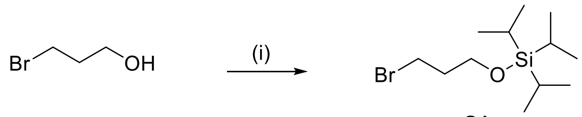

S1

Scheme S3. Reagents and conditions: (i) TIPSCl, imidazole, CH_2Cl_2, r.t., 1 h, quant.

S1

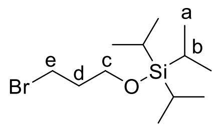

A solution of 3-bromo-1-propanol (1.8 mL, 20 mmol) in dry CH_2Cl_2 (40 mL) was treated with TIPSCl (4.3 mL, 20 mmol) and imidazole (3.4 g, 50 mmol). The solution was stirred at room temperature for 1 h. The reaction mixture was washed with H_2O (50 mL) and brine (50 mL), dried over $MgSO_4$, filtered and concentrated under reduced pressure to afford the desired compound as a colourless oil (5.9 g, quantitative). Characterisation data is in agreement with the literature.[2]

^{1}H NMR (600 MHz, $CDCl_3$) δ 3.82 (t, J = 5.7 Hz, 2H, c), 3.56 (t, J = 6.5 Hz, 2H, e), 2.06 (p, J = 6.1 Hz, 2H, d), 1.13 – 1.04 (m, 21H, a + b).

S2.5 Synthesis of macrocycle **1b**

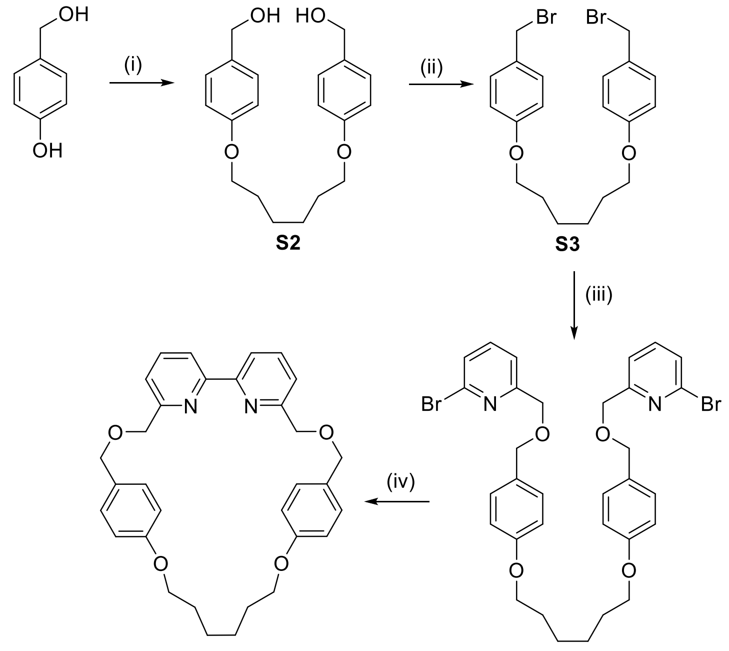

Scheme S4. Reagents and conditions: (i) 1,6-dibromohexane, K_2CO_3, MeCN, 90 °C, 24 h, 82%. (ii) conc. HBr, CH_2Cl_2, r.t., 3 h, 96%. (iii) NaH (60% in mineral oil), (6-bromo-pyridin-2-yl)methanol, DMF, 0 °C, then r.t., 1 h, 60%. (iv) $NiBr_2(PPh_3)_2$, Mn, Et_4NI, PPh_3, DMF, 50 °C, 1 h, then **S4**, 5 h, 50 °C, 55%.

S3

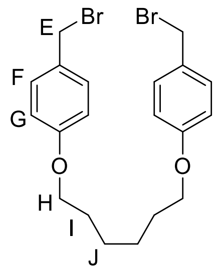

A suspension of 4-hydroxybenzyl alcohol (6.2 g, 50 mmol), 1,6-dibromohexane (3.9 mL, 25 mmol) and K_2CO_3 (17.3 g, 125 mmol) in MeCN (200 mL) was heated to 90 °C. After 12 h, more MeCN (25 mL) was added and stirring was continued for 12 h until the reaction was allowed to cool to room temperature. Solids were removed by filtration and the filtrate was concentrated to give a crude solid that was triturated in acetone (100 mL) and collected by filtration to give **S2** as a colourless solid (6.8 g, 82%) that was used in the next step without further purification. A solution of **S2** (6.8 g, 21 mmol) in CH_2Cl_2 (100 mL) was treated with conc. HBr (48%, 50 mL) and the biphasic mixture was vigorously stirred at room temperature for 3 h. The reaction mixture was diluted with water (100 mL), the layers were separated, and the aqueous layer was extracted with CH_2Cl_2 (3 x 60 mL). Combined organic layers were dried over Na_2SO_4, filtered and concentrated to give **S3** as a colourless solid (9.0 g,

96%) that was used in the next step without further purification. Characterisation data is in agreement with the literature.[3]

^{1}H NMR (600 MHz, $CDCl_3$) δ 7.31 (d, *J* = 8.6 Hz, 4H, F), 6.85 (d, *J* = 8.6 Hz, 4H, G), 4.51 (s, 4H, E), 3.96 (t, *J* = 6.4 Hz, 4H, H), 1.81 (dd, *J* = 8.4, 4.9 Hz, 4H, I), 1.54 (dd, *J* = 7.6, 3.9 Hz, 4H, J).

S4

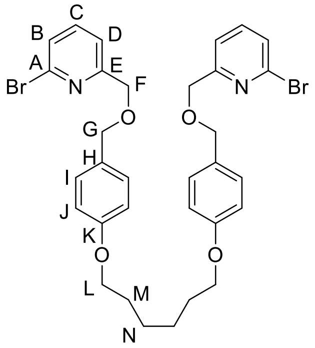

To a solution of **S3** (2.3 g, 5.0 mmol) in DMF (25 mL) at 0 °C was added NaH (660 mg, 60% dispersion in mineral oil, 16.5 mmol) portion-wise over 10 min under vigorous stirring, followed by the addition of (6-bromo-pyridin-2-yl)methanol (2.1 g, 11 mmol). The reaction mixture was stirred at room temperature for 1 h before H_2O (60 mL) was added. The suspension was filtered and the retained solid dissolved in $CHCl_3$ (40 mL). The organic layer was washed with brine (40 mL), dried over Na_2SO_4, filtered and concentrated under reduced pressure. Purification by flash chromatography (SiO_2, CH_2Cl_2:PE:EtOAc 11:11:2) afforded **S4** as a colourless solid (2.0 g, 60%).

^{1}H NMR (600 MHz, $CDCl_3$) δ 7.55 (t, *J* = 7.7 Hz, 2H, C), 7.46 (d, *J* = 7.6 Hz, 2H, D), 7.37 (d, *J* = 7.8 Hz, 2H, B), 7.28 (d, *J* = 8.6 Hz, 4H, I), 6.88 (d, *J* = 8.6 Hz, 4H, J), 4.62 (s, 4H, F), 4.57 (s, 4H, G), 3.97 (t, *J* = 6.5 Hz, 4H, L), 1.81 (p, *J* = 6.8 Hz, 4H, M), 1.55 – 1.51 (m, 4H, N). **^{13}C NMR** (151 MHz, $CDCl_3$) δ 160.66 (E), 159.04 (K), 141.37 (A), 139.11 (C), 129.69 (H), 129.68 (I), 126.67 (B), 120.09 (D), 114.60 (J), 72.94 (G), 72.09 (F), 68.00 (L), 29.34 (M), 26.02 (N); **M.p.** 77 – 81 °C; **HRMS** (ESI+) Calc. $C_{32}H_{34}N_2O_4Br_2K$ $[M+K]^+$ 707.0517, Obs. 707.0509.

1b

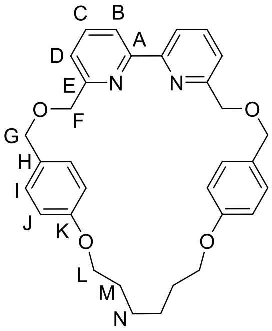

A flask was charged with $NiBr_2(PPh_3)_2$ (745 mg, 1.00 mmol), Mn (540 mg, 10.0 mmol), Et_4NI (207 mg, 1.00 mmol), PPh_3 (525 mg, 2.00 mmol) and DMF (10 mL). The suspension was sonicated for 10 min and subsequently heated to 50 °C for 1 h. **S4** (670 mg, 1.00 mmol) was added dropwise to the mixture as a solution in DMF (10 mL) over 4 h and after the addition had finished, stirring continued at 50 °C for 1 h. The suspension was allowed to cool to room temperature and CH_2Cl_2 (50 mL) and EDTA·NH_3 (50 mL) were added. The mixture was stirred for 5 min and then filtered through a pad of Celite®. The organic layer was washed with H_2O (2 x 50 mL), brine (50 mL), dried over Na_2SO_4, filtered and concentrated. PPh_3 was removed by filtration over a plug of silica (PE: CH_2Cl_2 1:1) and the fractions containing product were repurified by flash chromatography (SiO_2, PE: Et_2O 1:1) to yield **1b** as a colourless solid (282 mg, 55%).

^{1}H NMR (600 MHz, Chloroform-*d*) δ 7.77 (dd, *J* = 7.8, 1.2 Hz, 2H, B), 7.73 (t, *J* = 7.7 Hz, 2H, C), 7.44 (dd, *J* = 7.6, 1.1 Hz, 2H, D), 7.19 (d, *J* = 8.6 Hz, 4H, I), 6.76 (d, *J* = 8.6 Hz, 4H, J), 4.66 (s, 4H, G), 4.57 (s, 4H, F), 3.96 (t, *J* = 6.3 Hz, 4H, L), 1.76 (p, *J* = 6.7 Hz, 4H, M), 1.47 (p, *J* = 3.5 Hz, 4H, N); **^{13}C NMR** (151 MHz, $CDCl_3$) δ 159.31 (E), 158.99 (K), 156.17 (A), 137.28 (C), 130.31 (I), 129.79 (H), 121.02 (D), 120.53 (B), 114.89 (J), 72.79 (G), 71.68 (F), 67.99 (L), 28.79 (M), 25.41 (N); **M.p.** 122 – 124 °C; **HRMS** (ESI+) Calc. $C_{32}H_{34}N_2O_4Na$ $[M+Na]^+$ 533.2411, Obs. 533.2394.

S2.6 Synthesis of redox-active ester **2**

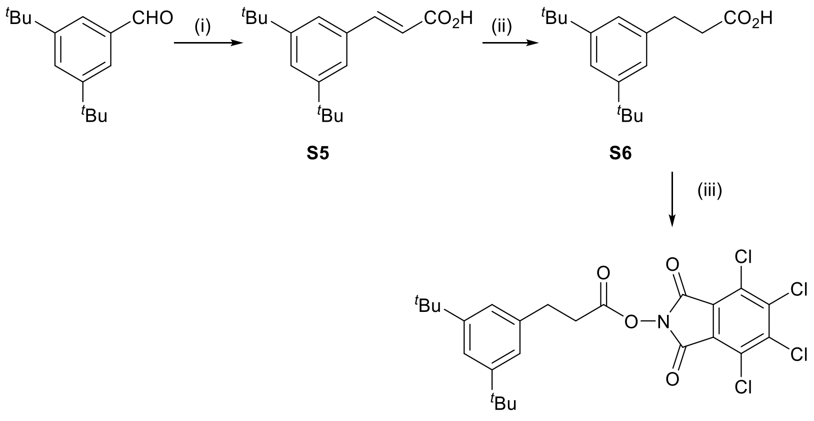

Scheme S5. Reagents and conditions: (i) 3,5-di-*tert*-butylbenzaldehyde, malonic acid, piperidine, pyridine, 120 °C, 3.5 h, quant. (ii) H_2, Pd/C, THF, r.t., 2 h, 98%. (iii) *N*-hydroxy-tetrachlorophthalimide, EDCI·HCl, DMAP, CH_2Cl_2, r.t., 2 h, 70%.

S6

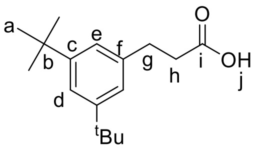

A solution of 3,5-di-*tert*-butylbenzaldehyde (1.4 g, 6.4 mmol) in pydridine (30 mL) was treated with malonic acid (1.0 g, 9.6 mmol) and heated to 120 °C. Piperidine (0.19 mL, 1.9 mmol) was added dropwise to the reaction. After 3 h, more malonic acid (0.5 g, 4.8 mmol) was added and stirring continued for 30 min. The reaction was allowed to cool to room temperature and diluted with 1 M HCl (100 mL). The pH was adjusted to pH = 1 with conc. HCl (ca. 15 mL) upon which a white solid precipitated. The aqueous layer was extracted with EtOAc (3 x 50 mL) and the combined organic layers were washed with brine (100 mL), dried over $MgSO_4$, filtered and concentrated to afford **S5** as a colourless solid (1.69 g, quantitative) that was used without further purification. A solution of **S5** (1.69 g, 6.4 mmol) in dry THF (25 mL) was treated with 10% palladium on activated carbon (0.17 g) and the solution was sparged with H_2 for 2 h at room temperature. The reaction mixture was filtered through a pad of Celite® and concentrated under reduced pressure to give **S6** as a colourless solid (1.65 g, 98%) that was used in the next step without further purification. Characterisation data is in agreement with the literature.[4]

^{1}H NMR (600 MHz, $CDCl_3$): δ 10.77 (br. s, 1H, j), 7.29 (t, *J* = 1.8 Hz, 1H, d), 7.06 (d, *J* = 1.8 Hz, 2H, e), 2.97 (dd, *J* = 9.0, 7.1 Hz, 2H, g), 2.74 – 2.67 (m, 2H, h), 1.32 (s, 18H, a).

2

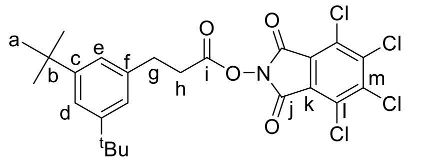

A solution of **S6** (1.28 g, 4.89 mmol) in dry CH_2Cl_2 (40 mL) was treated with *N*-hydroxytetrachloro-phthalimide (1.47 g, 4.89 mmol), EDCI·HCl (1.03 g, 5.37 mmol), and DMAP (60 mg, 0.45 mmol). The reaction was stirred at room temperature for 2 h before it was concentrated and purified by flash chromatography (SiO_2, *n*-hexane:EtOAc 19:1 to 9:1) to afford the desired compound as a colourless solid (1.87 g, 70%).

^{1}H NMR (600 MHz, $CDCl_3$) δ 7.32 (t, *J* = 1.8 Hz, 1H, d), 7.08 (d, *J* = 1.8 Hz, 2H, e), 3.09 (dd, *J* = 9.4, 6.7 Hz, 2H, g), 3.01 – 2.96 (m, 2H, h), 1.33 (s, 18H, a); **^{13}C NMR** (151 MHz, $CDCl_3$): δ 168.77 (i), 157.68 (j), 151.35 (c), 141.18 (k/l/m), 138.25 (f), 130.64(k/l/m), 124.86(k/l/m), 122.58 (e), 121.00 (d), 35.00 (b), 33.09 (h), 31.62 (a), 31.25 (g); **M.p.** 170 – 175 °C. **HRMS** (ESI-) Calc. $C_{26}H_{28}NO_5$ $[M+MeO]^-$ 576.0698, Obs. 576.0706.

S2.7 Synthesis of cross-coupling free thread **6**

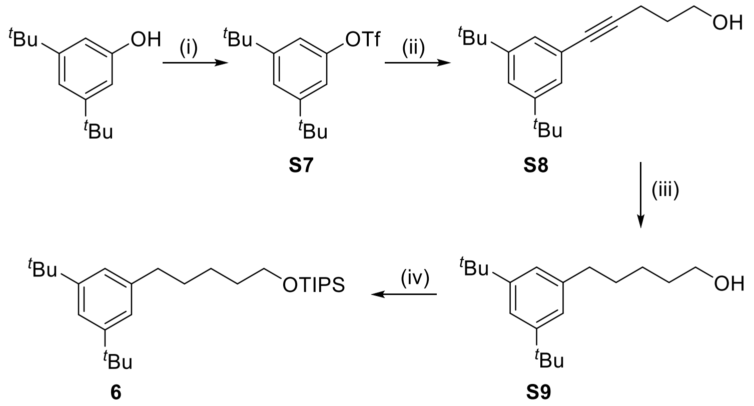

Scheme S6. Reagents and conditions: (i) Tf_2O, Et_3N, CH_2Cl_2, r.t., 4 h, quant. (ii) pent-4-yn-1-ol, $Pd(PPh_3)_2Cl_2$, CuI, Et_3N, THF, 80 °C, 14 h, 56%. (iii) H_2, Pd/C 20 h, then Pt/C 45 min, THF, r.t., quant. (iv) TIPSCl, imidazole, CH_2Cl_2, r.t., 20 h, 70%.

S7

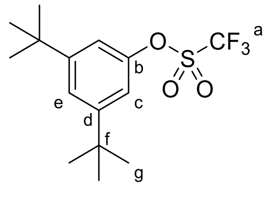

A solution of 3,5-di-*tert*-butylphenol (1.00 g, 4.85 mmol) in dry CH_2Cl_2 (20 mL) was cooled to 0 °C and NEt_3 (1.35 mL, 9.70 mmol) was added. Trifluoromethanesulfonic anhydride (1 M in

CH_2Cl_2, 5.8 mL, 5.8 mmol) was added dropwise and the reaction was allowed to warm to room temperature. After 3 h, more trifluoromethanesulfonic anhydride (1 M in CH_2Cl_2, 3.0 mL, 3.0 mmol) was added and the reaction was stirred for another 30 min. The mixture was washed with H_2O (25 mL), sat. aq. $NaHCO_3$ (25 mL) and brine (25 mL). The organic layer was dried over $MgSO_4$, filtered and concentrated to give a brown oil. Purification by flash chromatography (SiO_2, *n*-hexane) afforded compound **S7** as a colourless, low-melting solid (1.65 g, quantitative). Characterisation data was in agreement with the literature.[5]

^{1}H NMR (600 MHz, Chloroform-*d*) δ 7.41 (t, *J* = 1.6 Hz, 1H, e), 7.06 (d, *J* = 1.6 Hz, 2H, c), 1.32 (s, 18H, g).

S8

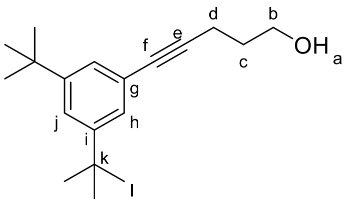

A flask was charged with **S7** (740 mg, 2.20 mmol), $Pd(PPh_3)_2Cl_2$ (154 mg, 0.220 mmol) and CuI (82 mg, 0.44 mmol). Solids were dissolved in dry degassed THF (10 mL), NEt_3 (4 mL) and pent-4-yn-1-ol (0.41 mL, 4.4 mmol) were added to the reaction and the mixture was heated at 80 °C for 14 h. The mixture was filtered through a pad of Celite® and concentrated under reduced pressure. The residue was taken up in EtOAc (25 mL) and washed with 1 M HCl (2 x 25 mL), sat. aq. $NaHCO_3$ (25 mL) and brine (25 mL). The organic layer was dried over $MgSO_4$, filtered and concentrated under reduced pressure. Purification by flash chromatography (SiO_2, PE:EtOAc 90:10 to 85:15) afforded compound **S8** as a low melting solid (338 mg, 56%).

^{1}H NMR (600 MHz, Chloroform-*d*) δ 7.34 (t, *J* = 1.9 Hz, 1H, j), 7.25 (d, *J* = 1.8 Hz, 2H, h), 3.84 (q, *J* = 5.9 Hz, 2H, b), 2.55 (t, *J* = 6.9 Hz, 2H, d), 1.88 (p, *J* = 6.7 Hz, 2H, c), 1.50 (t, *J* = 5.5 Hz, 1H, a), 1.30 (s, 18H, l). **^{13}C NMR** (151 MHz, $CDCl_3$) δ 150.84 (i), 125.91 (h), 122.69 (g), 122.32 (j), 87.98 (e), 82.29 (f), 62.13 (b), 34.91 (k), 31.59 (c), 31.47 (l), 16.19 (d). **HRMS** (ESI+) Calc. $C_{19}H_{28}ONa$ $[M+Na]^+$ 295.2032, Obs. 295.2030.

6

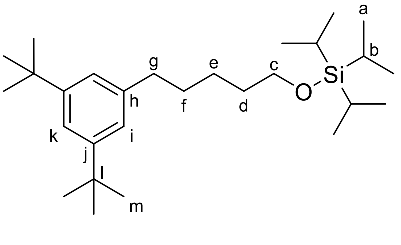

A solution of **S8** (328 mg, 1.20 mmol) in dry THF (10 mL) was charged with palladium on carbon (10% wt. loading, 35 mg). The mixture was sparged with hydrogen and stirred under

an atmosphere of hydrogen at room temperature for 20 h. Platinum on carbon (10% wt. loading, 30 mg) and dry THF (10 mL) were added to the mixture and the reaction was sparged with hydrogen for another 45 min. The mixture was filtered through a pad of Celite® and the organic layer was concentrated under reduced pressure to yield **S9** as a yellow oil (324 mg, quantitative) that was used in the next step without further purification. A solution of **S9** (300 mg, 1.08 mmol) in dry CH_2Cl_2 (5 mL) was charged with imidazole (188 mg, 2.71 mmol). TIPSCl (0.23 mL, 1.08 mmol) was added dropwise at room temperature and the reaction was stirred for 20 h. The mixture was diluted with CH_2Cl_2 (25 mL) and washed with H_2O (3 x 25 mL) and brine (25 mL). The organic layer was dried over $MgSO_4$, filtered and concentrated to give a yellow oil. Purification by flash chromatography (SiO_2, PE to PE:EtOAc 95:5) afforded **6** as a colourless oil (331 mg, 70%).

^{1}**H NMR** (600 MHz, $CDCl_3$) δ 7.25 (t, *J* = 1.8 Hz, 1H, k), 7.03 (d, *J* = 1.8 Hz, 2H, i), 3.69 (t, *J* = 6.6 Hz, 2H, c), 2.63 – 2.56 (m, 2H, g), 1.67 – 1.62 (m, 2H, f), 1.62 – 1.57 (m, 2H, d), 1.48 – 1.40 (m, 2H, e), 1.32 (s, 18H, m), 1.11 – 1.04 (m, 21H, a + b). ^{13}**C NMR** (151 MHz, $CDCl_3$) δ 150.59 (j), 142.02 (h), 122.71 (i), 119.76 (k), 63.55 (c), 36.82 (g), 34.90 (l), 33.06 (d), 31.88 (f), 31.67 (m), 26.03 (e), 18.21 (a), 12.16 (b). **HRMS** (ESI+) Calc. $C_{28}H_{52}OSiK$ $[M+K]^+$ 471.3419, Obs. 471.3425.

S2.8 Synthesis of homo-coupling free thread **7**

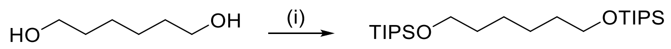

7

Scheme S7. Reagents and conditions: (i) TIPSCl, imidazole, CH_2Cl_2, r.t., 1 h, 96%.

7

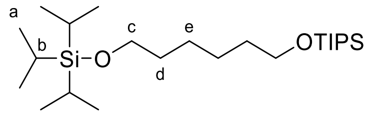

A solution of hexane-1,6-diol (0.59 g, 5.0 mmol) and imidazole (1.7 g, 25 mmol) in dry CH_2Cl_2 (10 mL) was treated with TIPSCl (2.1 mL, 10 mmol) and stirred at room temperature for 1 h. The mixture was diluted with CH_2Cl_2 (15 mL) and washed with H_2O (3 x 25 mL) and brine (25 mL). The organic layer was dried over $MgSO_4$, filtered and concentrated to give **7** as a colourless oil (2.1 g, 96%).

^{1}**H NMR** (600 MHz, $CDCl_3$) δ 3.67 (t, *J* = 6.7 Hz, 4H, c), 1.55 (m, 4H, d), 1.36 (m, 4H, e), 1.05 (m, 42H, a + b). ^{13}**C NMR** (151 MHz, $CDCl_3$) δ 63.58 (c), 33.19 (d), 25.82 (e), 18.19 (a), 12.16 (b). **HRMS** (ESI+) Calc. $C_{24}H_{54}O_2Si_2Na$ $[M+Na]^+$ 453.3560, Obs. 453.1657.

S2.9 Synthesis of rotaxanes **4a**, **4b**, **5a**, and **5b**

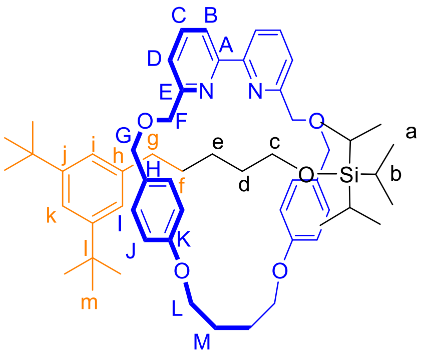

Rotaxane **4a** was synthesised according to general procedure **S2.2** (using 5.0 equivalents of **2** and 10.0 equivalents of **3**) in 19 % yield (determined by ^{1}H NMR).

^{1}H NMR (600 MHz, $CDCl_3$): δ 7.69 (t, J = 7.8 Hz, 2H, C), 7.59 (d, J = 7.7 Hz, 2H, B), 7.47 (d, J = 7.7 Hz, 2H, D), 7.21 (t, J = 1.9 Hz, 1H, k), 7.10 – 7.06 (m, 4H, I), 6.74 (d, J = 1.8 Hz, 2H, i), 6.63 (m, 4H, J), 4.64 – 4.53 (m, 4H, G), 4.42 (s, 4H, F), 4.05 – 3.92 (m, 4H, L), 2.99 – 2.92 (m, 2H, c), 2.06 – 1.86 (m, 4H, M), 1.65 – 1.57 (m, 2H, g), 1.34 (s, 18H, m), 1.06 – 0.96 (m, 21H, a-b), 0.57 (p, J = 8.0 Hz, 2H, d), 0.24 – 0.09 (m, 4H, e-f). **^{13}C NMR** (151 MHz, $CDCl_3$) δ 159.06 (E and K), 156.37 (A), 149.93 (j), 143.15 (h), 136.93 (C), 130.30 (I), 130.02 (H), 122.76 (i), 120.49 (B/D), 120.43 (B/D), 119.22 (k), 115.30 (J), 72.50 (G), 70.95 (F), 67.03 (L), 64.04 (c), 36.23 (g), 34.90 (l), 33.62 (d), 31.83 (m), 31.57 (e), 25.05 (M/f), 25.00 (M/f), 18.27 (a), 12.17 (b). **HRMS** (ESI+) Calc. $C_{58}H_{83}N_2O_5Si$ $[M+H]^+$ 915.6071, Obs. 915.6061.

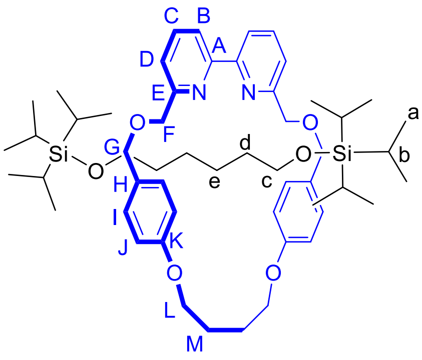

Rotaxane **5a** was synthesised according to general procedure **S2.2** (using 5.0 equivalents of **2** and 10.0 equivalents of **3**) in 20 % yield (determined by ^{1}H NMR).

^{1}H NMR (600 MHz, $CDCl_3$): δ 7.70 (t, J = 7.8 Hz, 2H, C), 7.55 (d, J = 7.7 Hz, 2H, B), 7.45 (d, J = 7.7 Hz, 2H, D), 7.21 – 7.16 (m, 4H, I), 6.81 – 6.76 (m, 4H, J), 4.63 (s, 4H, G), 4.37 (s, 4H, F), 4.02 – 3.96 (m, 4H, L), 3.03 – 2.96 (m, 4H, c), 1.96 – 1.88 (m, 4H, M), 1.06 – 0.90 (m, 42H, a-b), 0.60 (p, J = 7.7 Hz, 4H, d), 0.07 (p, J = 3.5 Hz, 4H, e). **^{13}C NMR** (151 MHz, $CDCl_3$): δ 159.19 (K), 159.00 (E), 156.38 (A), 136.85 (C), 130.34 (I), 130.04 (H), 120.47 (B), 120.32 (D), 115.47 (J), 72.59 (G), 70.91 (F), 67.10 (L), 63.97 (c), 33.34 (d), 24.94 (e), 24.90 (M), 18.27 (a), 12.14 (b). **HRMS** (ESI+) Calc. $C_{54}H_{85}N_2O_6Si_2$ $[M+H]^+$ 913.5946, Obs. 913.5936.

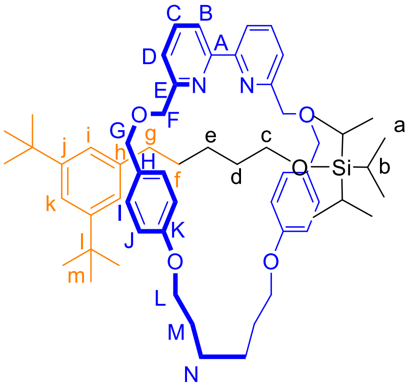

Rotaxane **4b** was synthesised according to general procedure **S2.2** (using 5.0 equivalents of **2** and 10.0 equivalents of **3**) in 38% yield (determined by ^{1}H NMR). The reaction was performed on a larger scale according to general procedure **S2.3**, affording 77 mg of a colourless oil (33% yield) after isolation.

^{1}H NMR (600 MHz, $CDCl_3$): δ 7.78 – 7.74 (m, 2H, B), 7.65 (t, *J* = 7.8 Hz, 2H, C), 7.49 – 7.45 (m, 2H, D), 7.20 (t, *J* = 1.9 Hz, 1H, k), 7.09 (d, *J* = 8.5 Hz, 4H, I), 6.72 (d, *J* = 1.8 Hz, 2H, i) 6.65 (d, *J* = 8.5 Hz, 4H, J), 4.63 (d, *J* = 2.3 Hz, 4H, G), 4.52 (s, 4H, F), 3.88 (td, *J* = 6.3, 4.4 Hz, 4H, L), 2.92 (t, *J* = 7.4 Hz, 2H, c), 1.75 – 1,77 (m, 4H, M), 1.60 – 1.55 (m, 2H, g), 1.43 (h, *J* = 4.1, 3.2, 4H, N), 1.35 (s, 18H, m), 1.05 – 0.95 (m, 21H, a-b), 0.51 (p, *J* = 7.5 Hz, 2H, d), 0.37 (p, *J* = 8.1 Hz, 2H,), 0.16 – 0.06 (m, 2H, e). **^{13}C NMR** (151 MHz, $CDCl_3$): δ 159.00 (E), 158.85 (K), 156.05 (A), 150.03 (j), 142.82 (h), 136.99 (C), 129.88 (H), 129.59 (I), 122.58 (i), 120.19 (D), 120.15 (B), 119.32 (k), 114.90 (J), 72.12 (G), 70.92 (F), 67.77 (L), 63.92 (c), 36.21 (g), 34.90 (l), 32.90 (d), 31.81 (m), 31.41 (f), 29.26 (M), 25.58 (N), 25.45 (e), 18.30 (a), 12.16 (b). **HRMS** (ESI+) Calc. $C_{60}H_{87}N_2O_5Si$ $[M+H]^+$ 943.6379, Obs. 943.6378.

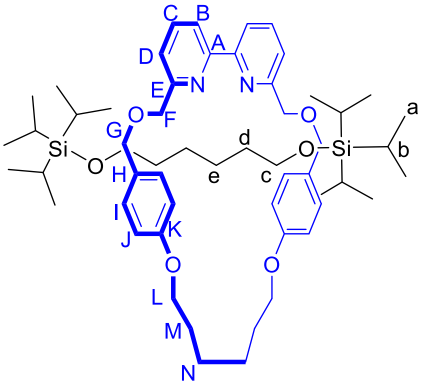

Rotaxane **5b** was synthesised according to general procedure **S2.2** (using 5.0 equivalents of **2** and 10.0 equivalents of **3**) in 18% yield (determined by ^{1}H NMR). The reaction was performed on a larger scale according to general procedure **S2.3**, affording 30 mg of a colourless oil (13% yield) after isolation.

^{1}H NMR (600 MHz, $CDCl_3$): δ 7.75 (dd, *J* = 7.8, 1.1 Hz, 2H, B), 7.70 (t, *J* = 7.7 Hz, 2H, C), 7.48 (dd, *J* = 7.6, 1.1 Hz, 2H, D), 7.16 – 7.12 (m, 4H, I), 6.75 – 6.71 (m, 4H, J), 4.64 (s, 4H, G), 4.49 (s, 4H, F), 3.91 (t, *J* = 6.4 Hz, 2H, L), 2.96 (t, *J* = 7.4 Hz, 2H, c), 1.74 – 1.69 (m, 4H, M), 1.41 (t, *J* = 3.5 Hz, 2H, N), 1.11 – 0.96 (m, 42H, a-b), 0.63 – 0.55 (m, 4H, d), 0.05 (td,

J = 7.8, 7.0, 3.1 Hz, 4H, e). **^{13}C NMR** (151 MHz, $CDCl_3$): δ 158.99 (E), 158.93 (K), 156.07 (A), 136.96 (C), 129.88 (H), 129.59 (I), 120.11 (B and D), 114.99 (J), 72.17 (G), 70.87 (F), 67.87 (L), 63.85 (c), 33.06 (d), 29.26 (M), 25.49 (N), 25.04 (e), 18.30 (a), 12.16 (b). **HRMS** (ESI+) Calc. $C_{56}H_{88}N_2O_6Si_2$ $[M+H]^+$ 941.6254, Obs. 941.6253.

S3. ^{1}H NMR Stackplots of Rotaxanes

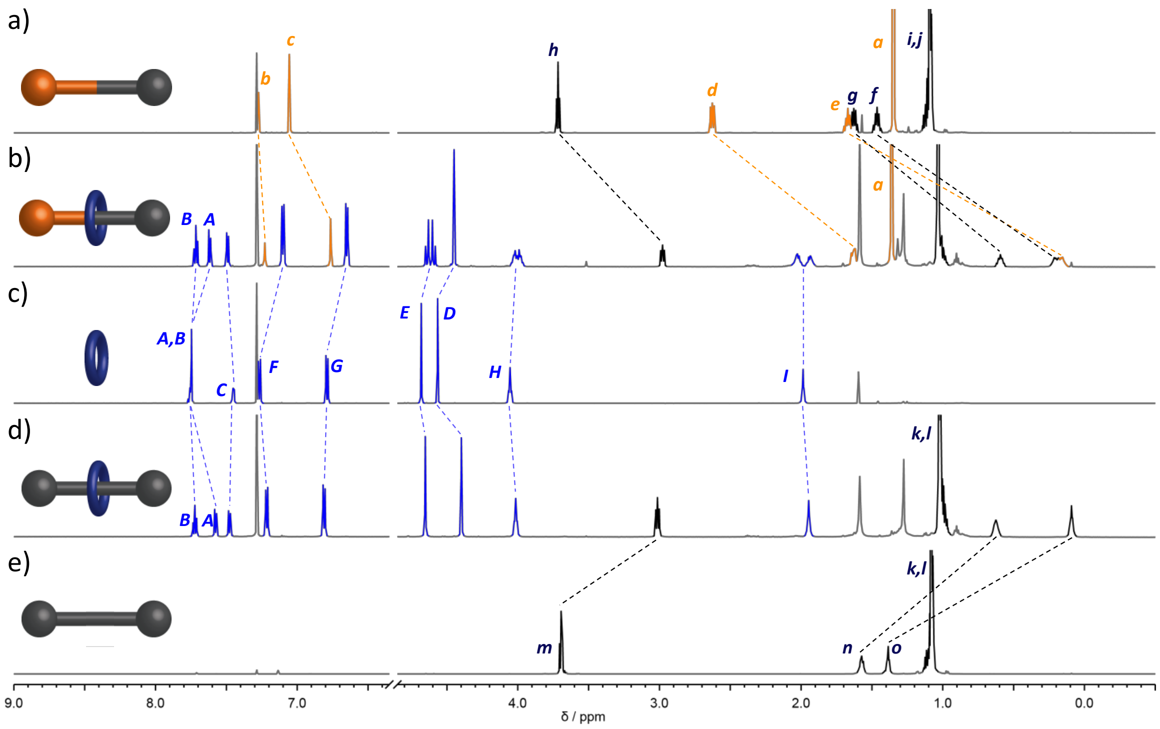

Figure S1: ^{1}H NMR spectra (600 MHz, $CDCl_3$, 298 K) of (a) unsymmetrical thread **6**, (b) rotaxane **4a**, (c) macrocycle **1a**, (d) rotaxane **5a**, and (e) symmetrical thread **7**. Assignments correspond to the labelling shown in Scheme 1 in main text of paper.

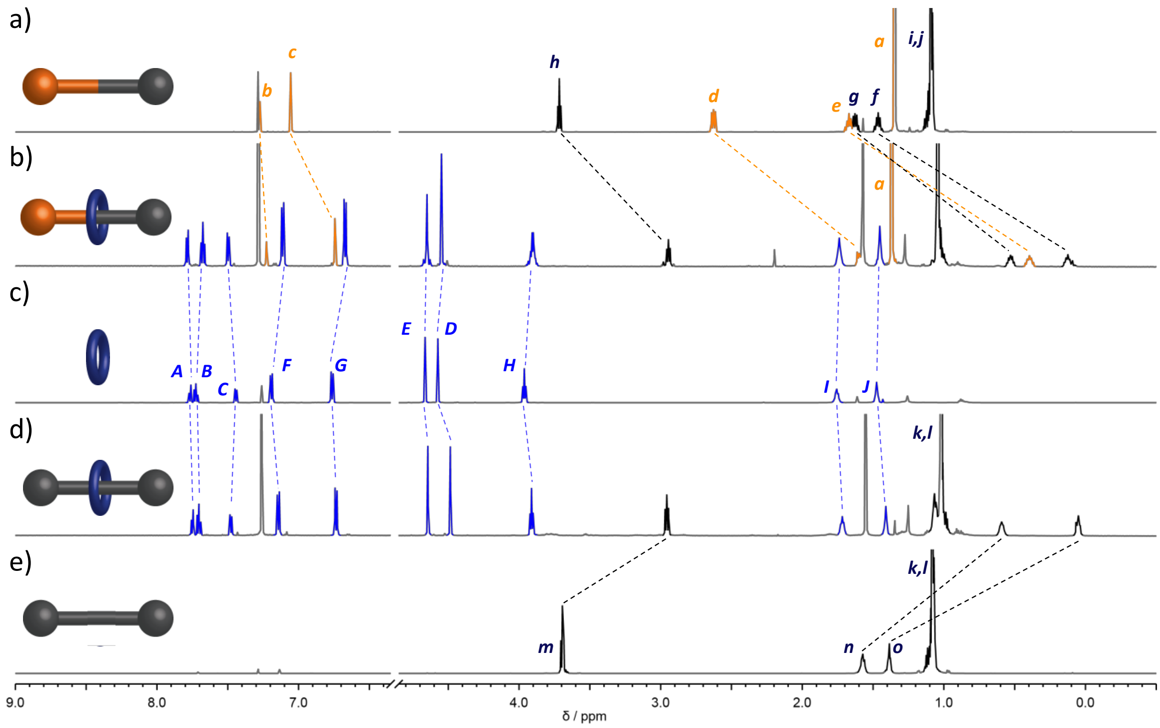

Figure S2: ^{1}H NMR spectra (600 MHz, $CDCl_3$, 298 K) of (a) unsymmetrical thread **6**, (b) rotaxane **4b**, (c) macrocycle **1a**, (d) rotaxane **5b**, and (e) symmetrical thread **7**. Assignments correspond to the labelling shown in Scheme 1 in main text of paper.

S4. ^{1}H and ^{13}C NMR Spectra

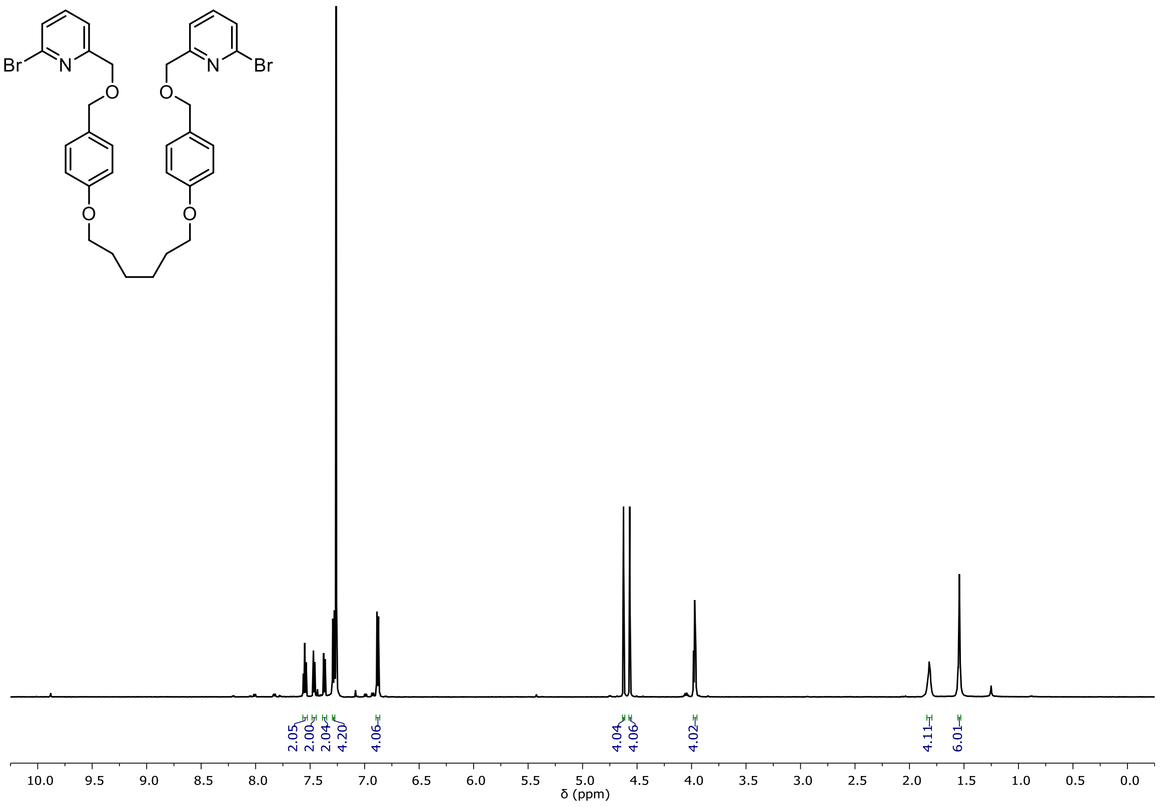

Figure S3: ^{1}H NMR (600 MHz, $CDCl_3$) spectrum of **S4**.

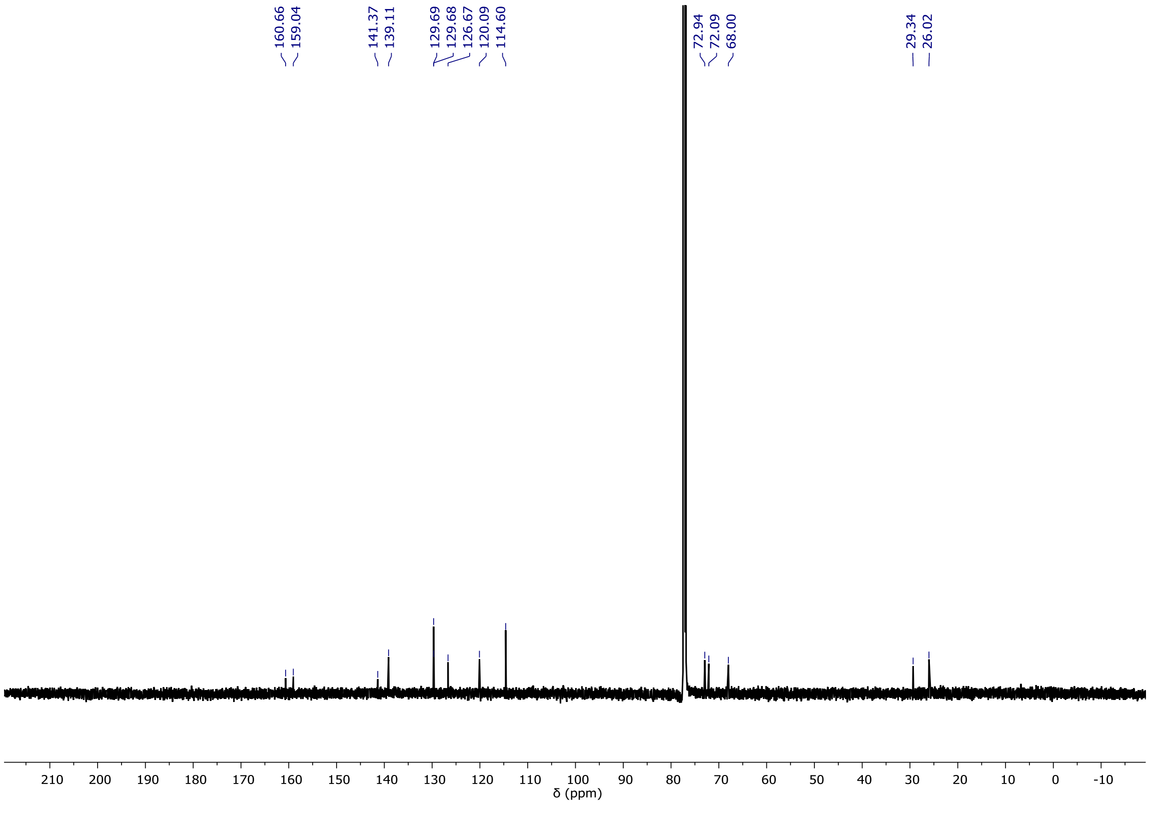

Figure S4: ^{13}C NMR (151 MHz, $CDCl_3$) spectrum of **S4**.

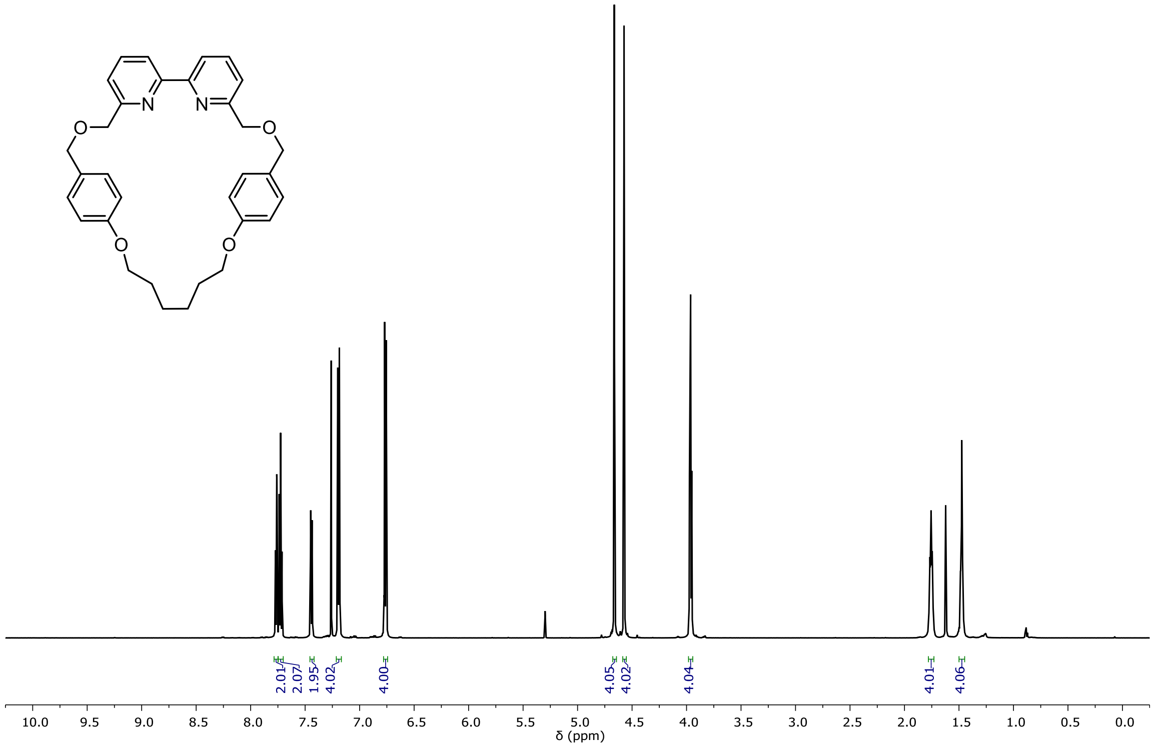

Figure S5: ^{1}H NMR (600 MHz, $CDCl_3$) spectrum of **1b**.

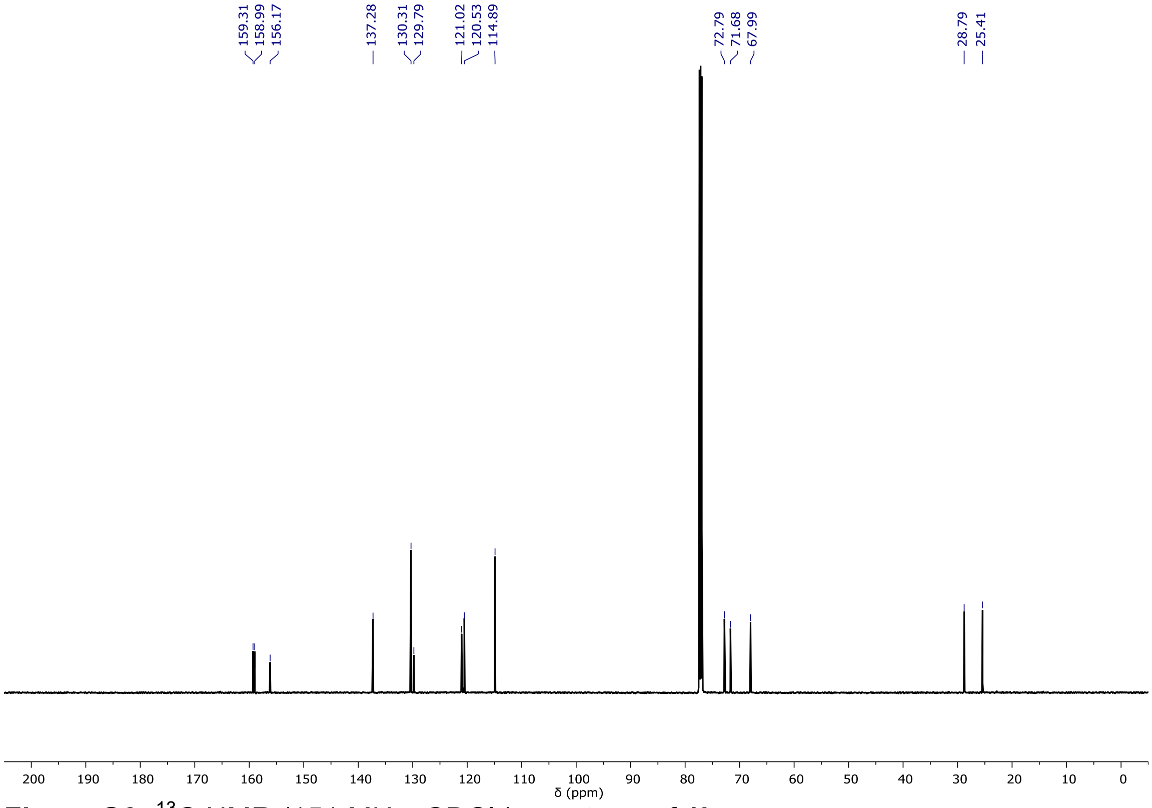

Figure S6: ^{13}C NMR (151 MHz, $CDCl_3$) spectrum of **1b**.

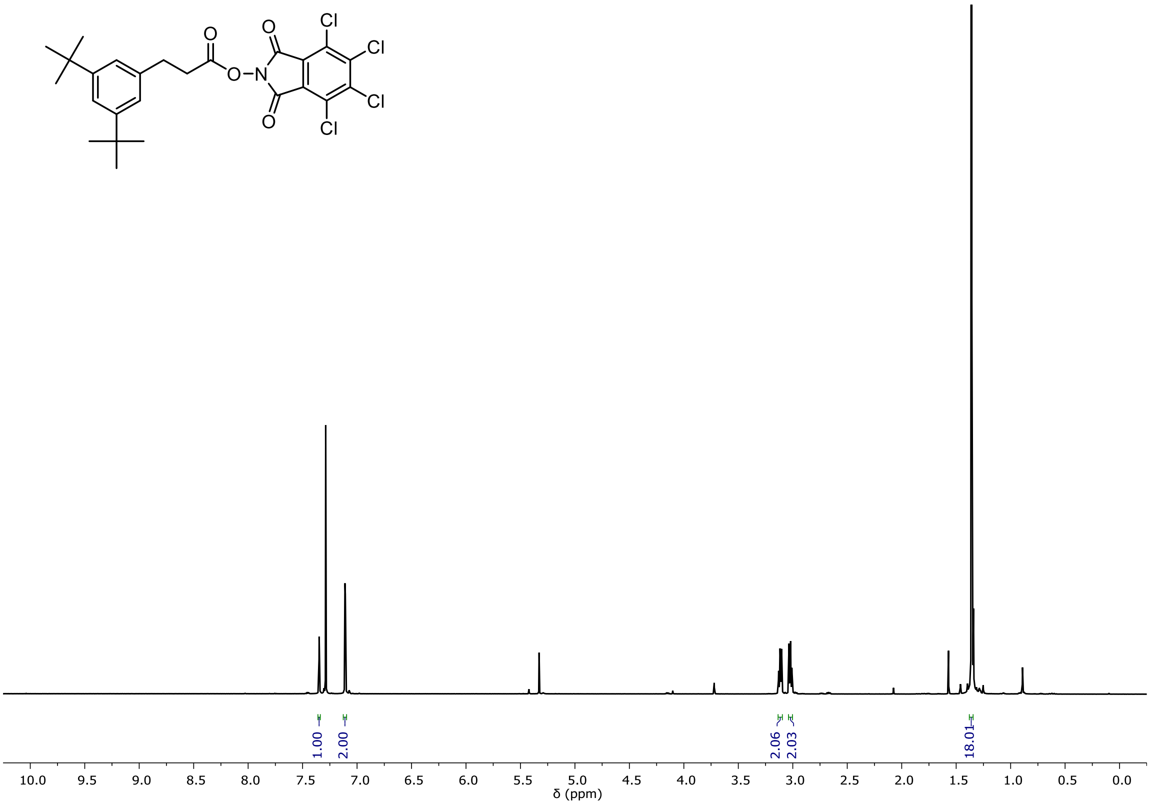

Figure S7: ^{1}H NMR (600 MHz, $CDCl_3$) spectrum of **2**.

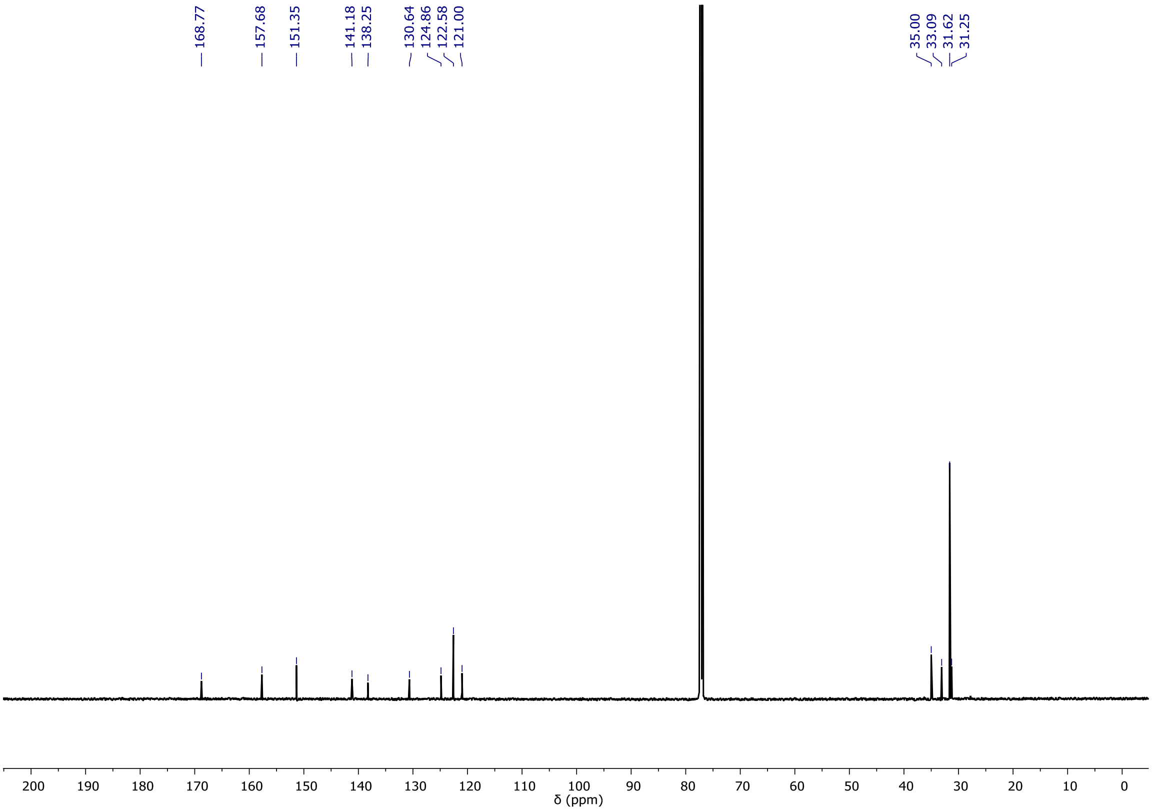

Figure S8: ^{13}C NMR (151 MHz, $CDCl_3$) spectrum of **2**.

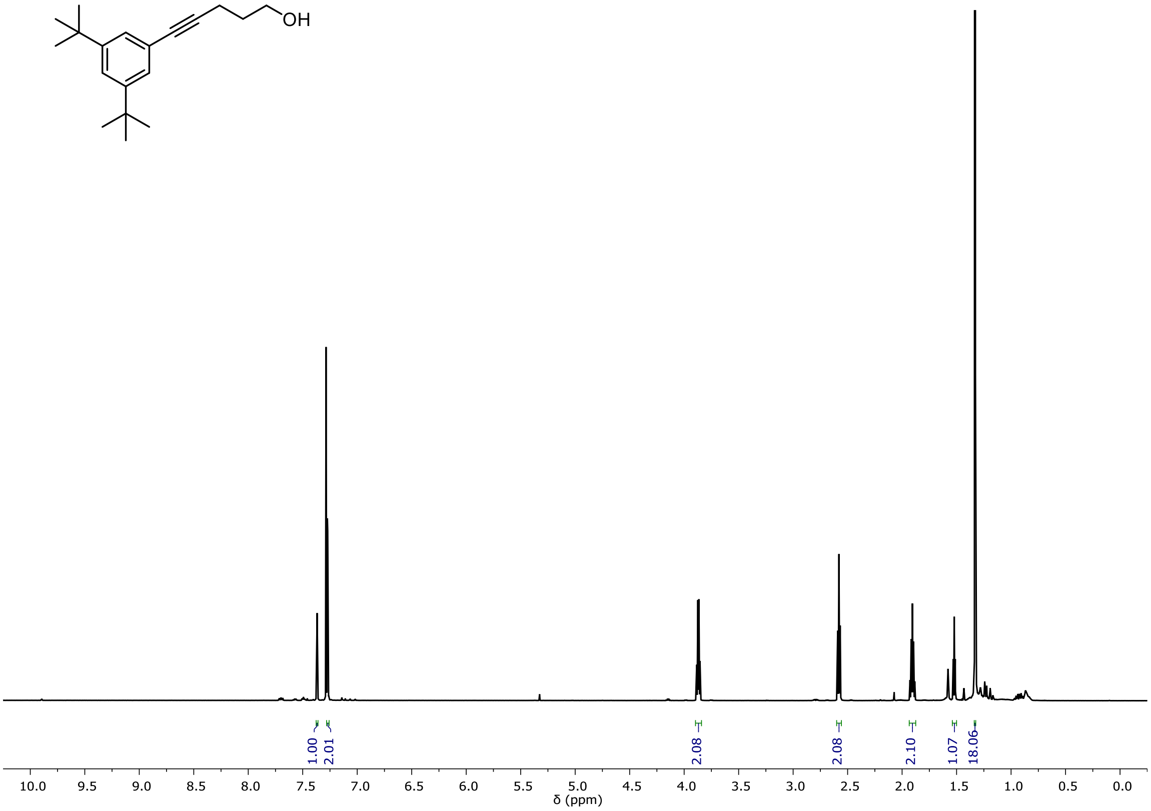

Figure S9: ^{1}H NMR (600 MHz, $CDCl_3$) spectrum of **S8**.

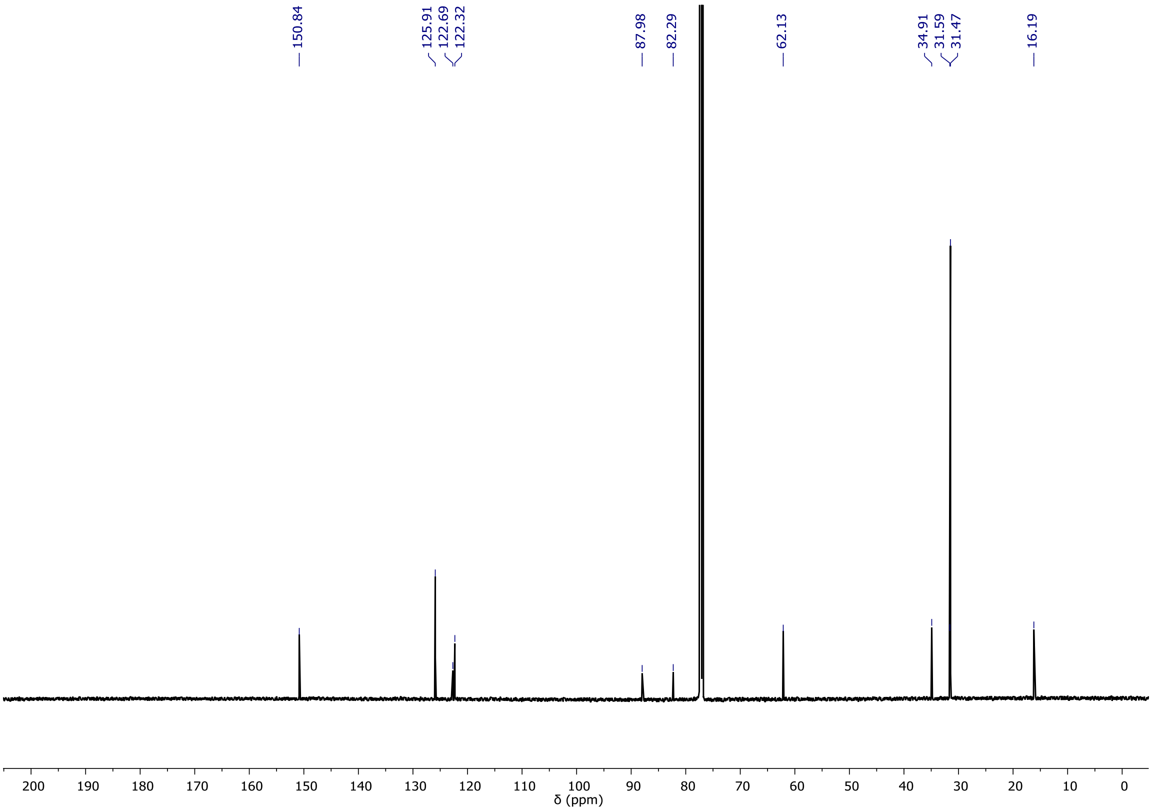

Figure S10: ^{13}C NMR (151 MHz, $CDCl_3$) spectrum of **S8**.

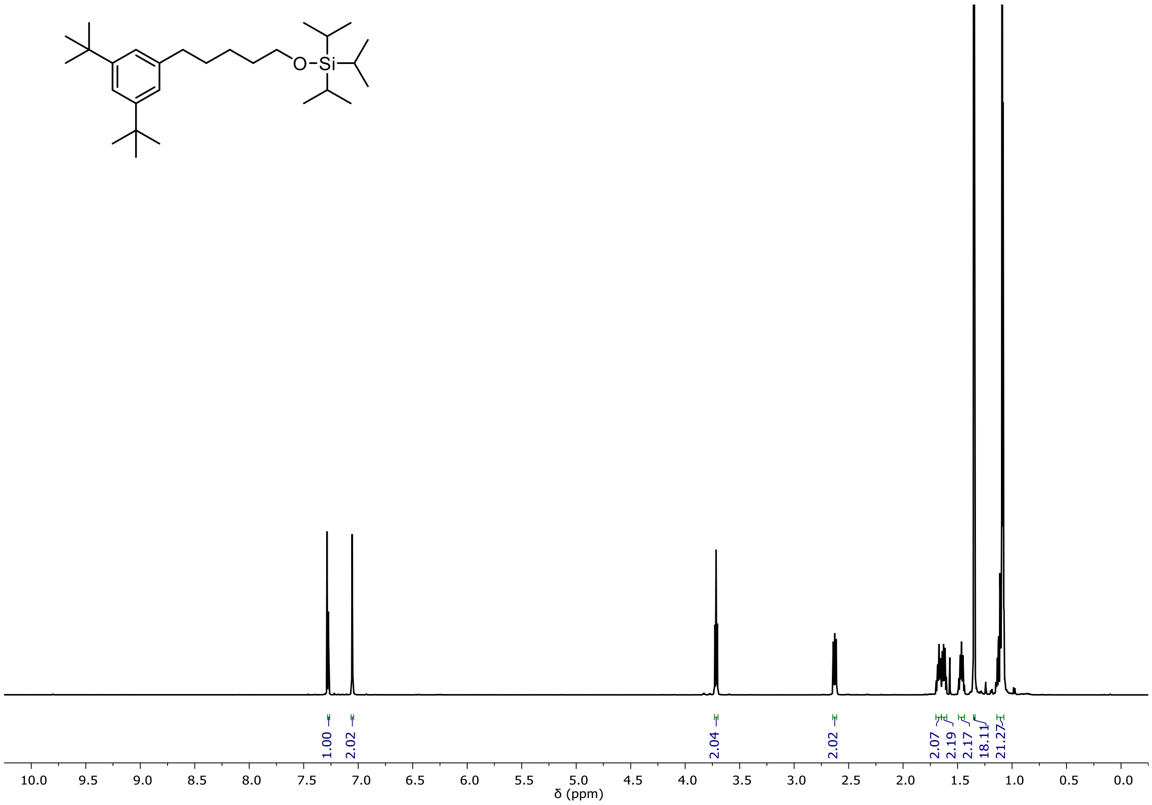

Figure S11: ^{1}H NMR (600 MHz, $CDCl_3$) spectrum of **6**.

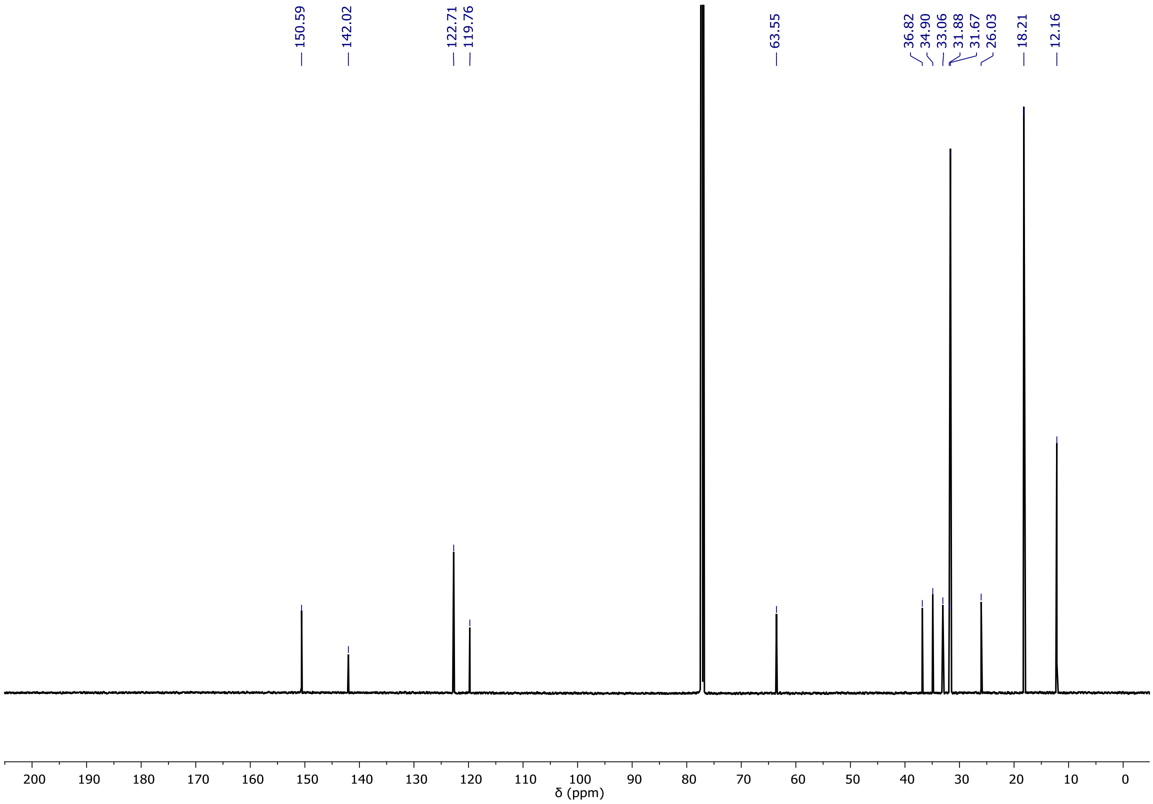

Figure S12: ^{13}C NMR (151 MHz, $CDCl_3$) spectrum of **6**.

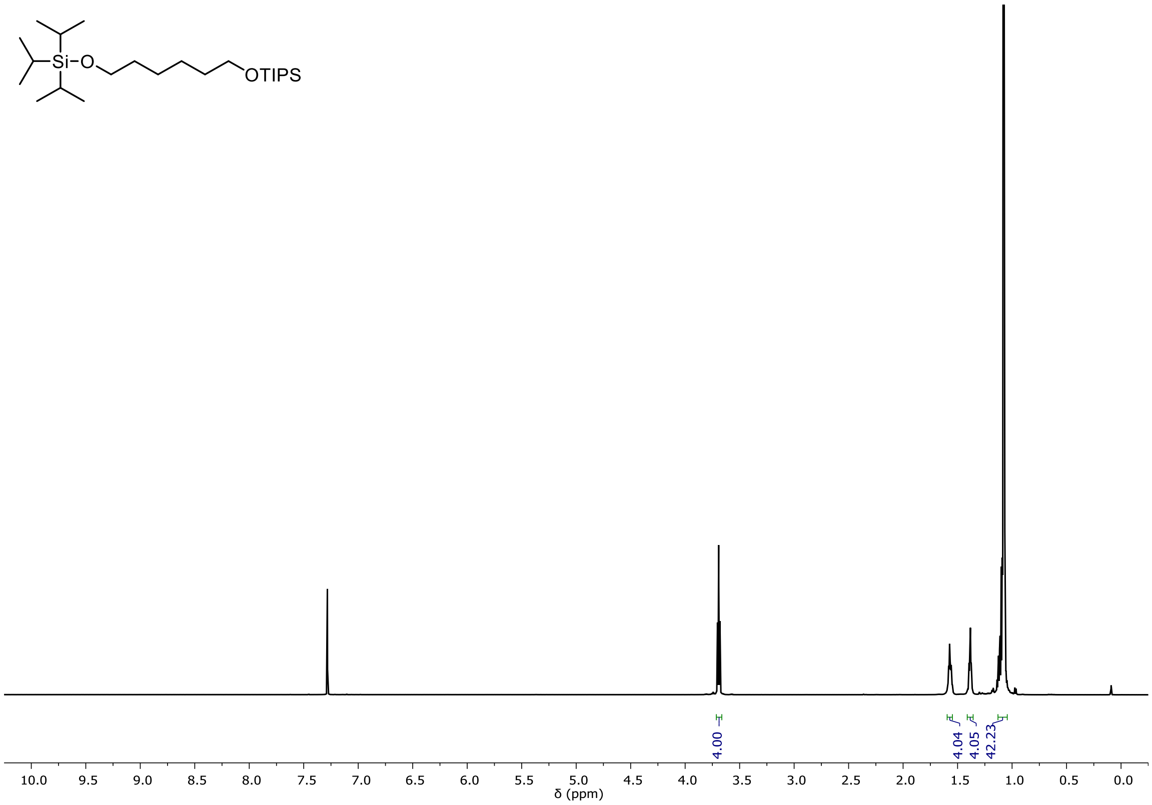

Figure S13: ^{1}H NMR (600 MHz, $CDCl_3$) spectrum of **7**.

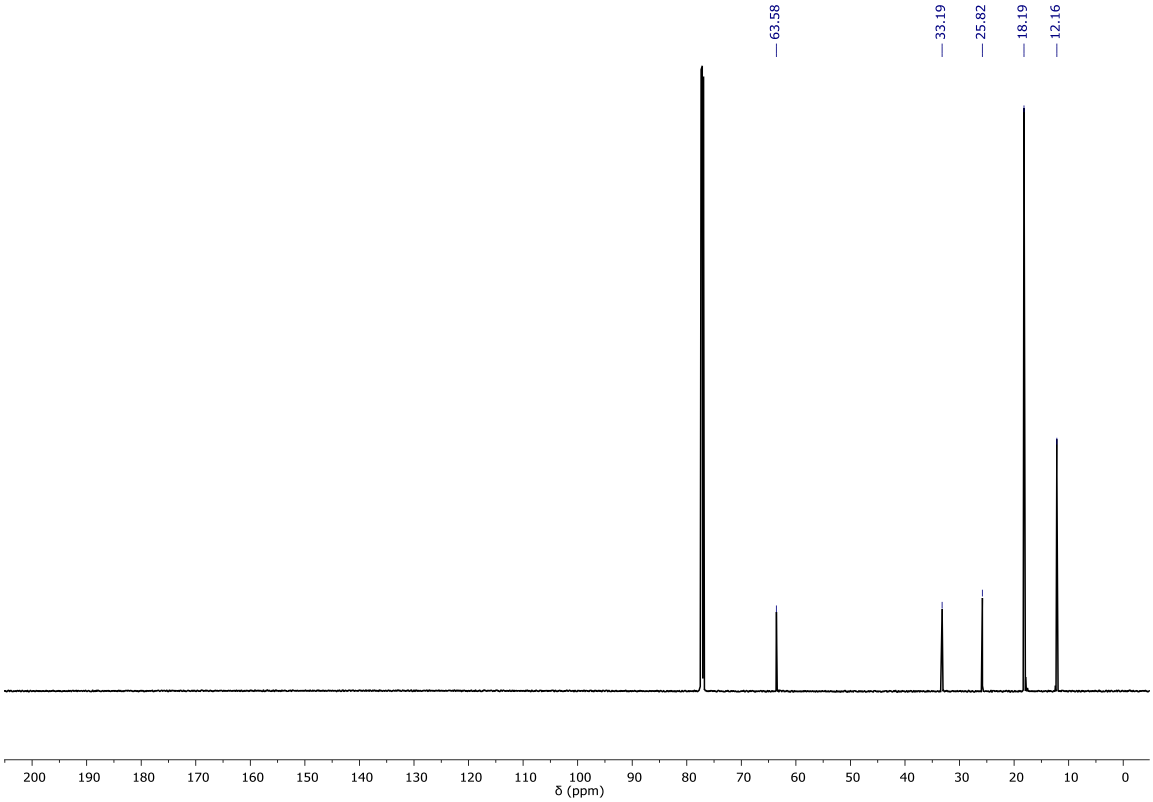

Figure S14: ^{13}C NMR (151 MHz, $CDCl_3$) spectrum of **7**.

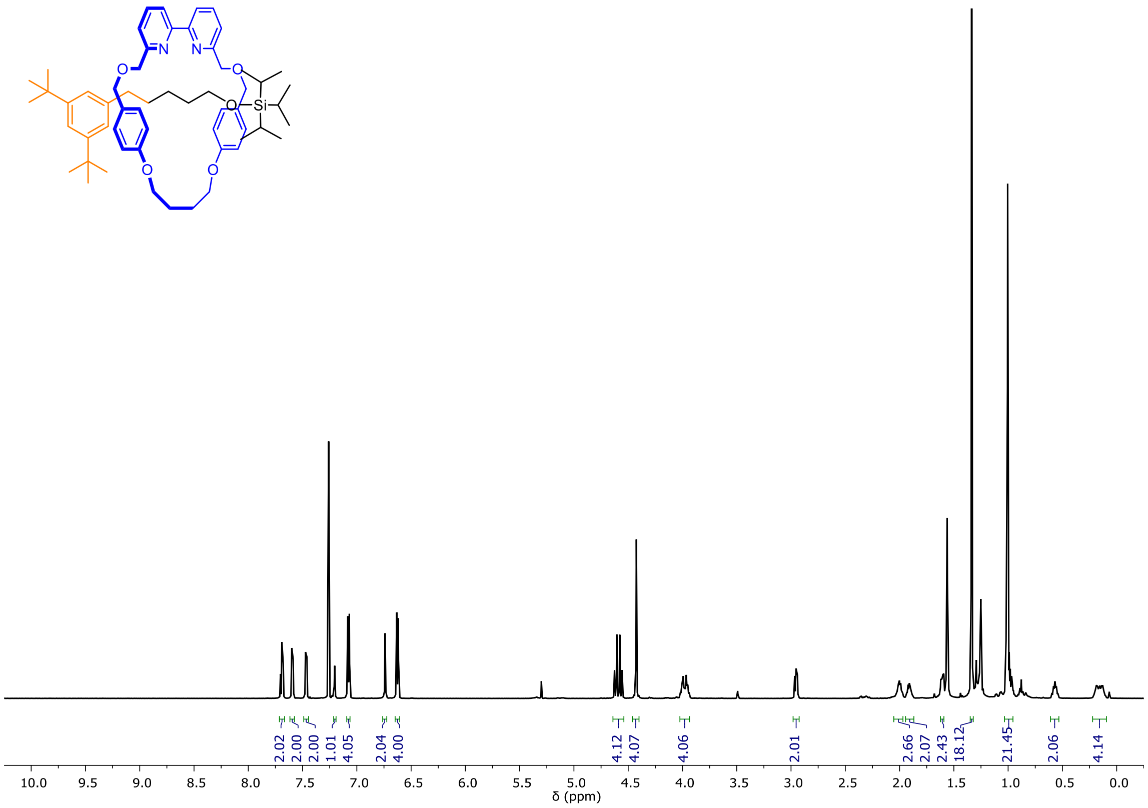

Figure S15: ^{1}H NMR (600 MHz, $CDCl_3$) spectrum of **4a**.

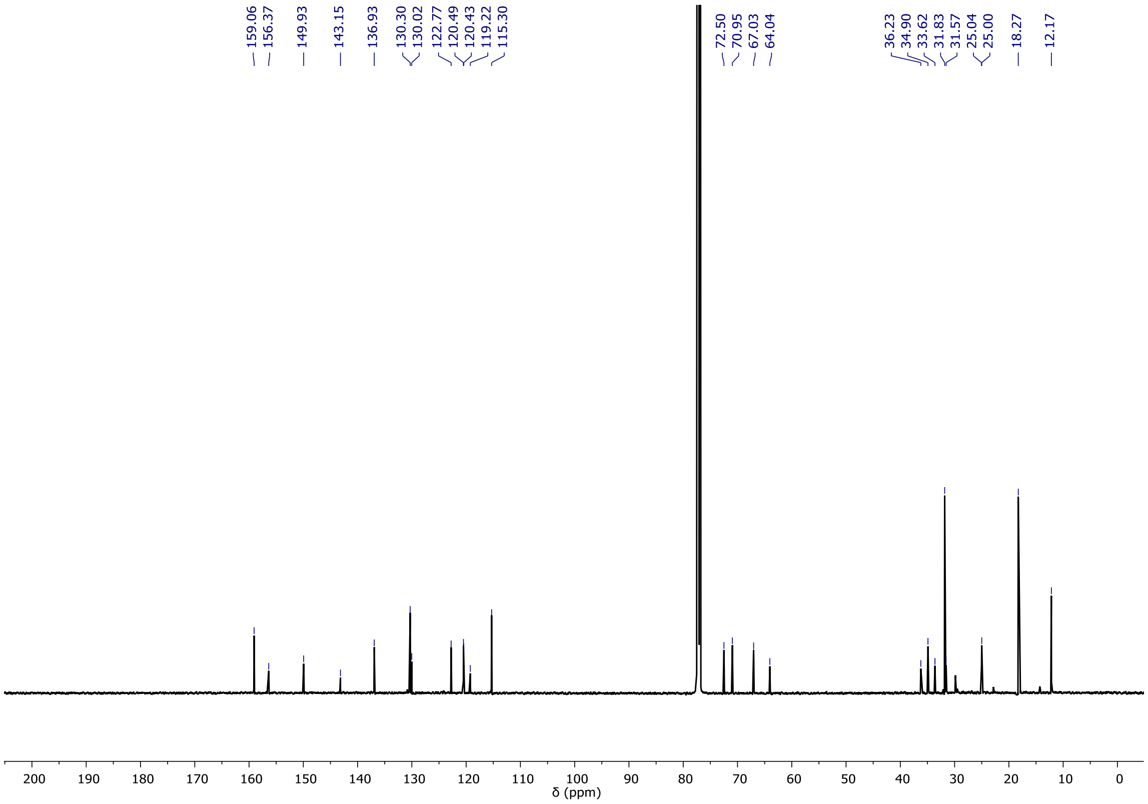

Figure S16: ^{13}C NMR (151 MHz, $CDCl_3$) spectrum of **4a**.

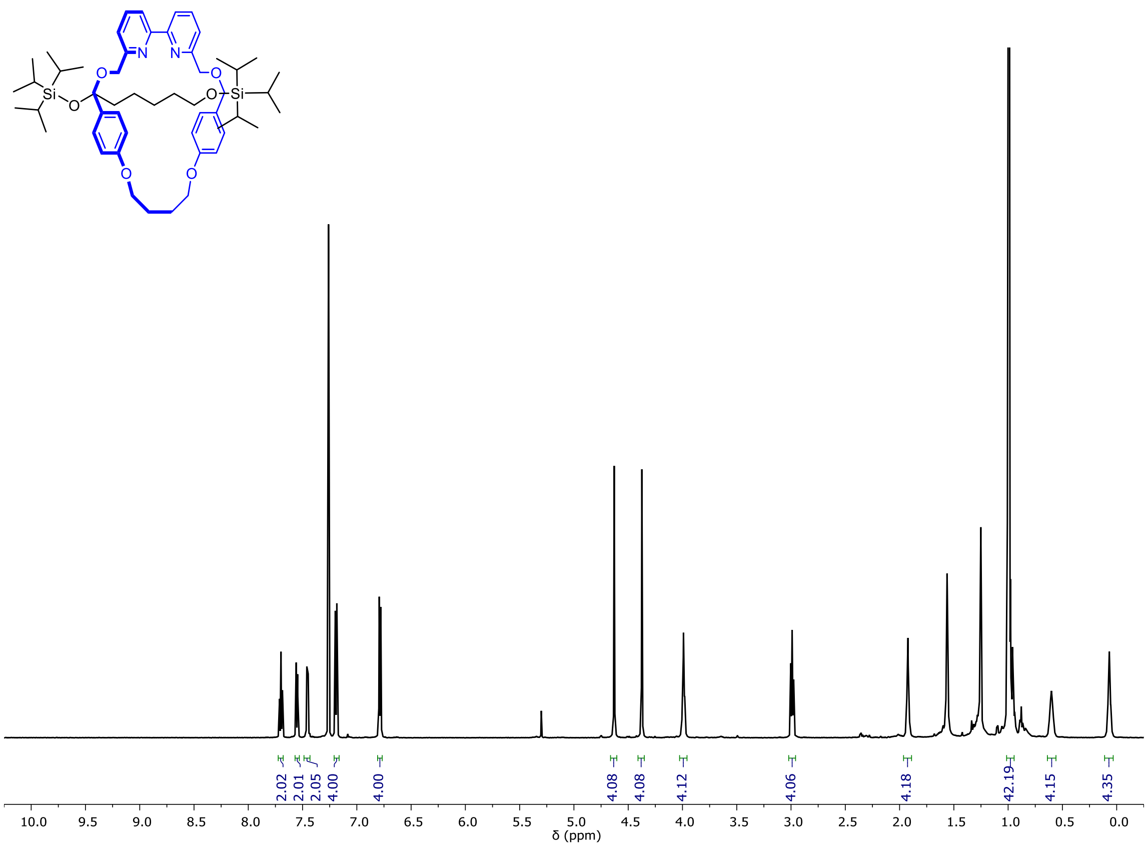

Figure S17: ^{1}H NMR (600 MHz, $CDCl_3$) spectrum of **5a**.

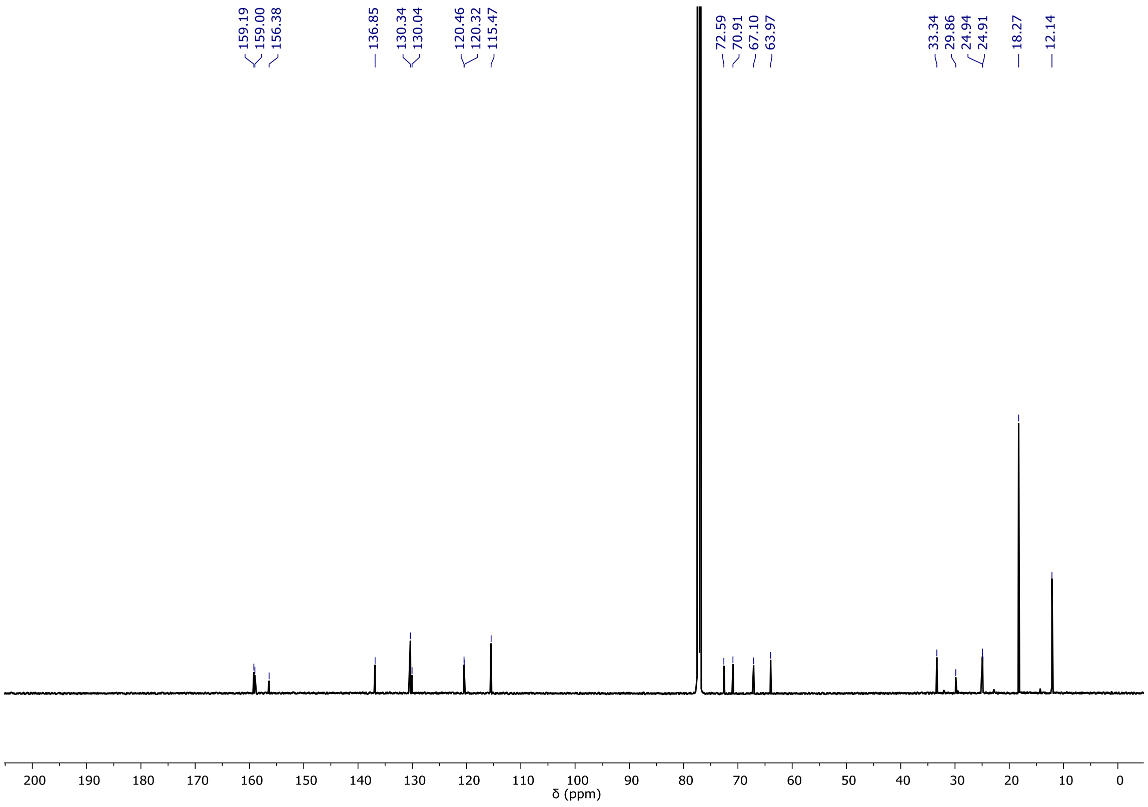

Figure S18: ^{13}C NMR (151 MHz, $CDCl_3$) spectrum of **5a**.

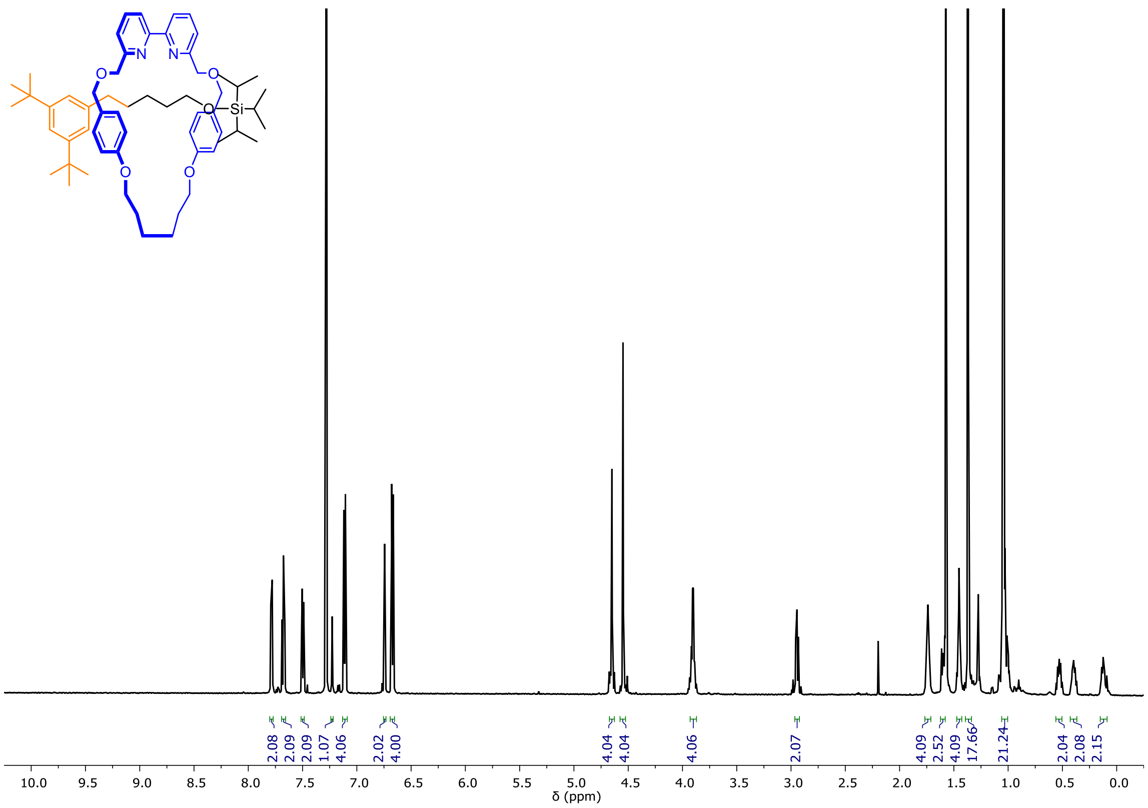

Figure S19: ^{1}H NMR (600 MHz, $CDCl_3$) spectrum of **4b**.

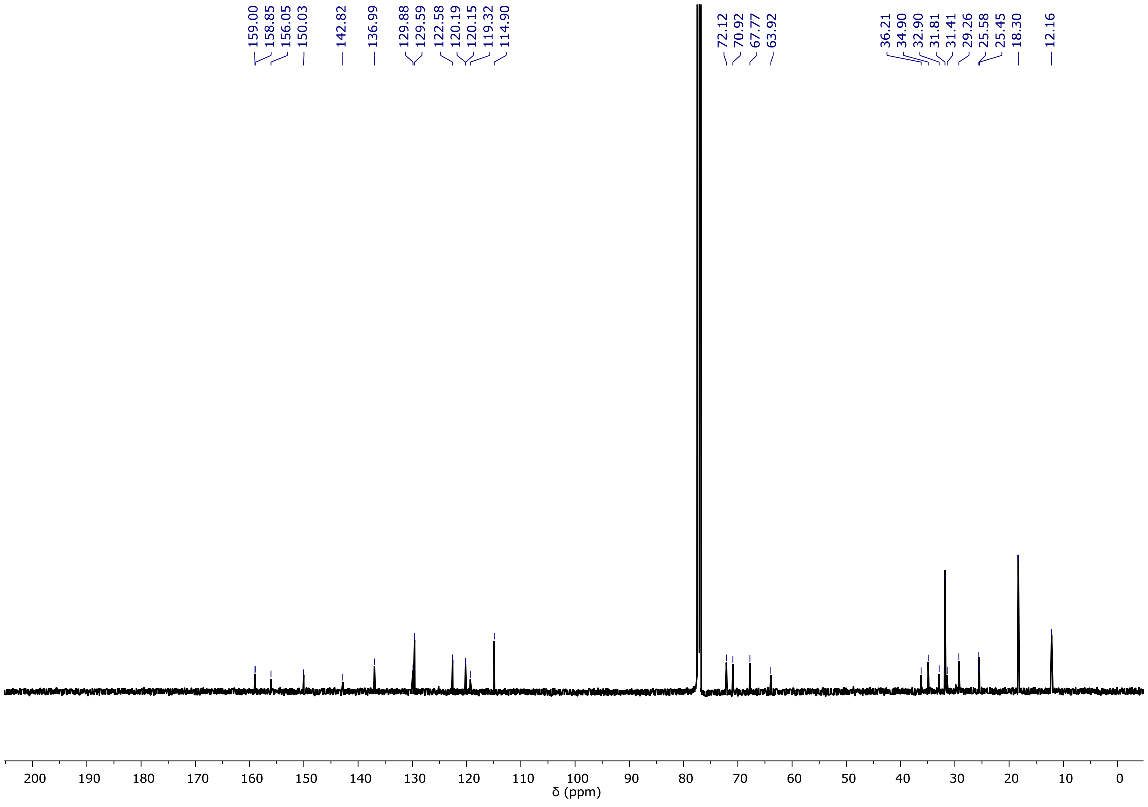

Figure S20: ^{13}C NMR (151 MHz, $CDCl_3$) spectrum of **4b**.

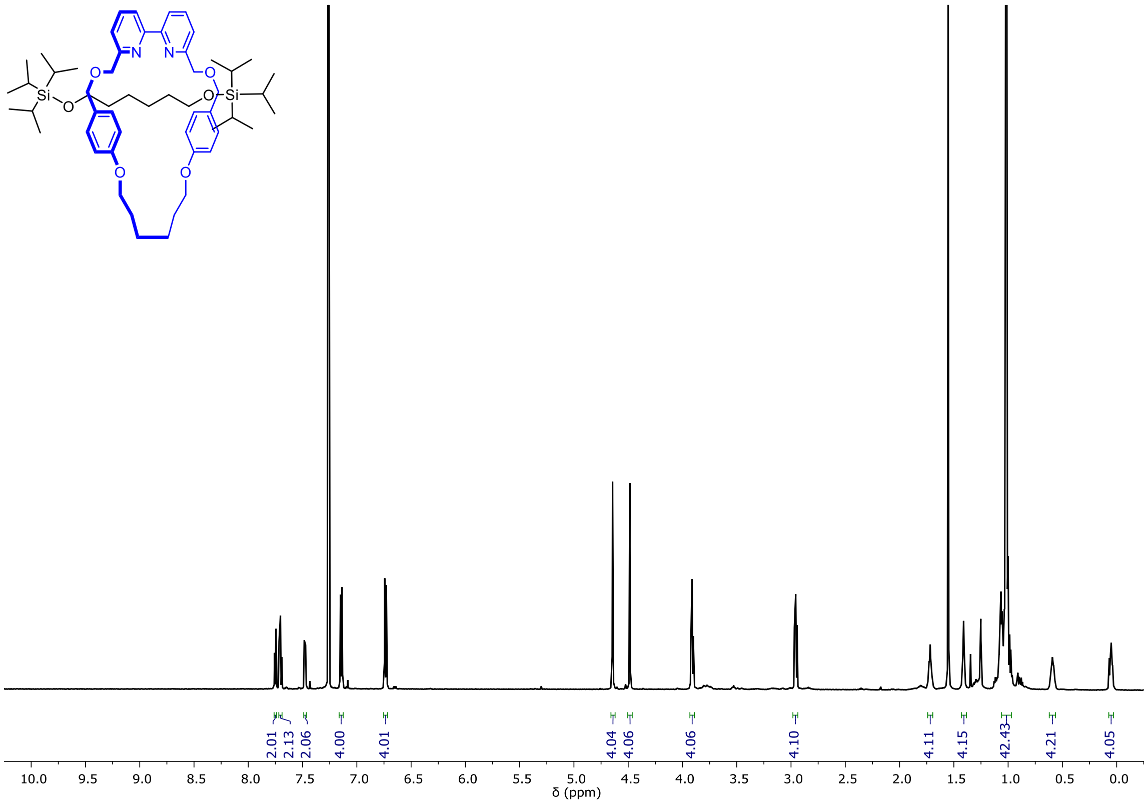

Figure S21: ^{1}H NMR (600 MHz, $CDCl_3$) spectrum of **5b**.

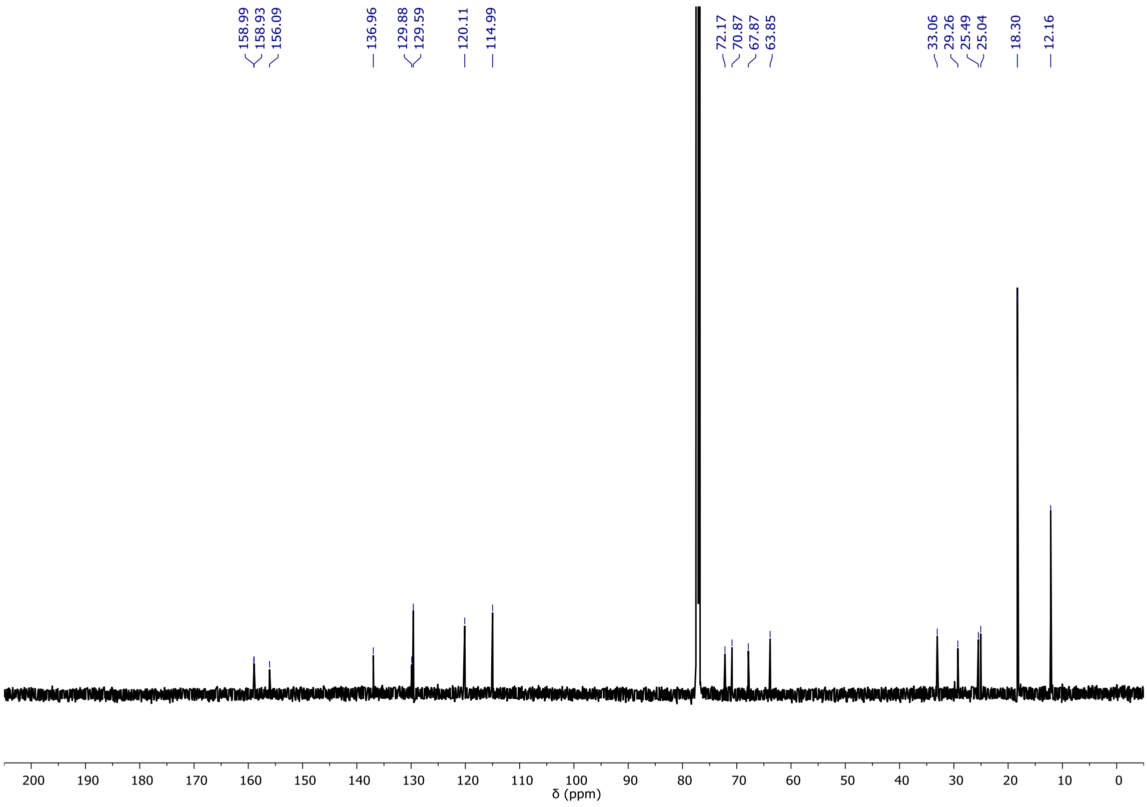

Figure S22: ^{13}C NMR (151 MHz, $CDCl_3$) spectrum of **5b**.

S5. References

1. J. E. M. Lewis, R. J. Bordoli, M. Denis, C. J. Fletcher, M. Galli, E. A. Neal, E. M. Rochette and S. M. Goldup, *Chem. Sci.,* 2016, **7**, 3154–3161.

2. J. Clayden, F. E. Knowles and I. R. Baldwin, *J. Am. Chem. Soc.,* 2005, **127**, 2412–2413.

3. C. A. Schalley, G. Silva, C. F. Nising and P. Linnartz, *Helv. Chim. Acta.,* 2002, **85**, 1578–1596.

4. F.-N. Li, N.-J. Kim, S.-M. Paek, D.-Y. Kwon, K. H. Min, Y.-S. Jeong, S.-Y. Kim, Y.-H. Park, H.-D. Kim, H.-G. Park and Y.-G. Suh, *Bioorg. Med. Chem.,* 2009, **17**, 3557–3567.

5. Z. Peng, Y. Pan, B. Xu and J. Zhang, *J. Am. Chem. Soc.,* 2000, **122**, 6619–6623.
